# High cumulative glucocorticoid dose is associated with increased levels of inflammation-related mediators in active rheumatoid arthritis

**DOI:** 10.3389/fimmu.2024.1505615

**Published:** 2024-12-18

**Authors:** Anna Petrackova, Pavel Horak, Jakub Savara, Martina Skacelova, Eva Kriegova

**Affiliations:** ^1^ Department of Immunology, Faculty of Medicine and Dentistry, Palacký University Olomouc and University Hospital, Olomouc, Czechia; ^2^ Department of Internal Medicine III - Nephrology, Rheumatology and Endocrinology, Faculty of Medicine and Dentistry, Palacký University Olomouc and University Hospital, Olomouc, Czechia; ^3^ Department of Computer Science, Faculty of Electrical Engineering and Computer Science, Technical University of Ostrava, Ostrava, Czechia

**Keywords:** autoimmune diseases, adverse effects of glucocorticoids, systemic inflammation, cytokine profile, long-treated patients, disease activity, serum protein pattern, blood cell count

## Abstract

**Introduction:**

Glucocorticoids (GCs) are widely used as a treatment for rheumatoid arthritis (RA), leading to high cumulative doses in long-term treated patients. The impact of a high cumulative GC dose on the systemic inflammatory response in RA remains poorly understood.

**Methods:**

We investigated long-treated patients with RA (n = 72, median disease duration 14 years) through blood counts and the serum levels of 92 inflammation-related proteins, and disease activity was assessed using the Simple Disease Activity Index (SDAI). Patients were grouped based on the cumulative GC dose, with a cut-off value of 20 g (low/high, n = 49/23).

**Results and discussion:**

Patients with a high cumulative GC dose within the active RA group had elevated serum levels in 23 inflammation-related proteins compared with patients with a low dose (cytokines/soluble receptors: CCL3, CCL20, CCL25, IL-8, CXCL9, IL-17A, IL-17C, IL-18, sIL-18R1, IL-10, sIL-10RB, OSM and sOPG; growth factors: sTGFα and sHGF; other inflammatory mediators: caspase 8, STAMBP, sCDCP1, sirtuin 2, 4E-BP1, sCD40, uPA and axin-1; p_corr_ < 0.05). In non-active RA, the high and low GC groups did not differ in analysed serum protein levels. Moreover, patients with active RA with a high GC dose had an increased white blood cell count, increased neutrophil–lymphocyte and platelet–lymphocyte ratios and a decreased lymphocyte–monocyte ratio compared with the low dose group (p < 0.05). This is the first study to report elevated serum levels in inflammation-related proteins and deregulated blood counts in patients with active RA with a high cumulative GC dose. The elevated systemic inflammation highlights the importance of improving care for patients receiving high cumulative GC doses.

## Introduction

Glucocorticoids (GCs) are widely used for the treatment of rheumatoid arthritis (RA), a disease characterised by inflammatory joint involvement with synovial hyperplasia, bone erosion and progressive function loss, accompanied by pain, morning stiffness and a limited range of joint movement (1). The goal of treatment with GCs and other modalities in RA is to reduce disease activity and achieve clinical remission in the short term and limit structural progression, disability and systemic manifestations in the long term (2).

GCs have an immunomodulatory effect acting at multiple levels in the pathogenesis of RA. Specifically, GCs were revealed to reduce the activation of monocytes/macrophages, T cells, eosinophils and basophils and downregulate circulating adhesion molecules and prostaglandins (3). Moreover, GCs polarise active pro-inflammatory macrophages (M1) towards anti-inflammatory ones (M2) in RA (3). From a clinical perspective, serious adverse effects associated with GC treatment, such as osteoporosis and metabolic disorders including diabetes type II, skin atrophy, cataracts and hypertension, have been recognised, and this has led to the recommendation that GCs should only be used for short periods (4, 5). The long-term adverse effects of GCs may be related to the high cumulative dose. Therefore, GCs should be dosed with a balance between efficacy and long-term safety in mind (2).

Although GCs have been used in the treatment of RA for more than 70 years, there is limited knowledge of how the long-term administration of high doses of GCs affects the systemic inflammatory response. Therefore, this study aimed to investigate the impact of a high cumulative GC dose on the systemic inflammation response in RA by analysing the serum profile of proteins associated with inflammation and blood counts in a real-world cohort of patients with RA receiving long-term treatment.

## Methods

### Study population and materials

Serum samples were obtained from 72 patients with RA who met the 2010 ACR/EULAR classification criteria for RA (1); the patients were recruited at a single tertiary rheumatology centre. All the patients were receiving long-term treatment (median disease duration 14 years) according to standard protocols (5, 6); for the medications used, disease duration, blood counts and demographic and clinical characteristics, see **Table 1**. Disease activity was assessed using the Simple Disease Activity Index (SDAI), with SDAI > 11 taken as active RA (non-active/active RA: 24/48). For a comprehensive assessment, we also incorporated the 28-joint Disease Activity Score using C-reactive protein (DAS28), with DAS28 > 3.2 taken as active RA (non-active/active RA: 35/37). The control group of healthy participants consisted of 25 age- and gender-matched individuals; subjects with the presence of inflammatory autoimmune diseases in first- or second-degree relatives, recent vaccinations, infections or who were using immunosuppressive drugs were excluded following completion of a questionnaire.

**Table 1:** Clinical characteristics of the rheumatoid arthritis patient cohort.

	Active RA			Non-active RA		
	High GC dose (n = 18)	Low GC dose (n = 30)	p value	High GC dose (n = 5)	Low GC dose (n = 19)	p value
Female/Male	16/2	22/8	0.192	3/2	17/2	0.133
Cumulative GC dose (g), median (min–max)	36.8 (21.0–150.0)	8.1 (0.75–18.0)	**< 0.0001**	36.0 (22.5–36.0)	5.4 (0.9–18.0)	**0.0008**
SDAI, median (min–max)	20.0 (11.5–38.7)	19.5 (11.6–53.0)	0.882	6.4 (4.1–8.6)	7.7 (1.4–11.0)	0.213
Age at the onset of the disease (yrs), median (min–max)	36.5 (16.0–57.0)	42.0 (15.0–60.0)	**0.034**	32.0 (5.0–55.0)	40.0 (10.0–57.0)	0.337
Duration of the disease (yrs), median (min–max)	21.5 (6.0–58.0)	8.5 (2.0–38.0)	**< 0.0001**	25.0 (16.0–28.0)	14.0 (1.0–21.0)	**0.004**
Osteoporosis, % (n)	72.2% (13)	43.3% (13)	**0.054**	100% (5)	26.3% (5)	**0.004**
HAQ, median (min–max)	2.0 (0.5–2.7)	0.8 (0–2.5)	**< 0.0001**	1.5 (0.125–2)	0.5 (0–2.25)	0.107
ESR (mm/hr), median (min–max)	23 (2–60)	18 (2–45)	0.197	3 (2–16)	13 (2–40)	0.073
CRP (mg/l), median (min–max)	6.9 (1.5–55.0)	5.1 (0.6–44)	0.083	0.6 (0.6–3.9)	1.9 (0.6–10.8)	0.080
ACPA (IU/ml), median (min–max)	227 (25–2475)	580 (25–3200)	0.096	1775 (25–3200)	306 (25–3200)	0.293
RF (IU/ml), median (min–max)	57.6 (9.8–644.0)	73.5 (10.0–728.0)	0.131	40.0 (10.3–146.0)	36.0 (9.8–330.0)	0.972
WBC count (10^9^/l), median (min–max)	8.6 (5.1–19.8)	6.8 (3.4–20.1)	**0.004**	5.6 (3.3–8.6)	5.9 (4.7–10.4)	0.814
Lymphocytes (10^9^/l), median (min–max)*	1.69 (0.72–4.14)	2.18 (0.44–3.51)	0.284	2.34 (1.06–2.75)	1.98 (1.20–4.74)	0.699
Monocytes (10^9^/l), median (min–max)*	0.83 (0.60–1.53)	0.58 (0.33–0.98)	**0.002**	0.61 (0.42–0.79)	0.55 (0.25–0.80)	0.832
Neutrophils (10^9^/l), median (min–max)*	5.67 (2.59–15.7)	3.81 (2.30–15.9)	0.138	3.54 (1.75–4.90)	3.20 (2.26–3.96)	0.945
Platelets (10^9^/l), median (min–max)	274 (196–475)	258 (128–588)	0.136	205 (166–258)	267 (202–392)	**0.047**
LMR, median (min–max)*	1.75 (0.95–4.70)	3.53 (0.96–6.58)	**0.001**	3.12 (2.52–5.48)	3.70 (2.12–5.93)	0.635
NLR, median (min–max)*	4.67 (0.63–7.51)	1.96 (0.94–5.23)	**0.030**	1.72 (0.91–2.29)	1.70 (0.70–2.49)	0.839
PLR, median (min–max)*	174.0 (47.3–387.2)	118.5 (43.6–522.7)	**0.022**	97.2 (63.1–173.6)	142.2 (67.9–298.3)	0.145
Medications, % (n)						
Glucocorticoids	94.4% (17)	76.7% (23)	0.115	80% (4)	57.9% (11)	0.374
Methotrexate	77.8% (14)	83.3% (25)	0.640	100% (5)	84.2% (16)	0.352
Other DMARDs	33.3% (6)	26.7% (8)	0.630	0% (0)	5.3% (1)	0.607
Biologics	44.4% (8)	43.3% (13)	0.941	60.0% (3)	42.1% (8)	0.484
NSAIDs	83.3% (15)	60.0% (18)	0.095	100% (5)	52.6% (10)	0.057

ACPA, anti-citrullinated protein antibody; CRP, C-reactive protein; DMARDs, disease-modifying anti-rheumatic drugs; ESR, erythrocyte sedimentation rate; GC, glucocorticoid; HAQ, Health Assessment Questionnaire Disability Index; LMR, lymphocyte–monocyte ratio; NLR, neutrophil–lymphocyte ratio; NSAIDs, non-steroidal anti-inflammatory drugs; PLR, platelet–lymphocyte ratio; RF, rheumatoid factor; SDAI, Simplified Disease Activity Index; WBC, white blood cell. *Data available for 64% (46/72) of patients. The p values reaching significance are in bold.

The patients and control subjects provided written informed consent for the use of their peripheral blood in this study in accordance with the Helsinki Declaration. The study was approved by the Ethics Committee of the University Hospital and Palacký University Olomouc (approval number NV15-28659A).

### Proximity extension immunoassay

The serum levels of 92 inflammation-related proteins were simultaneously measured using the Olink Inflammation kit I (Olink Bioscience, Sweden), as reported previously (7, 8). Of the 92 proteins analysed (**Supplementary Table S1**), the levels of 18 proteins, including TNFα and IL-1α, were below the limit of detection and were therefore excluded from further analysis.

### Statistical analysis

All statistical analyses were performed on linearised data (linear ddCq) for each analyte. The statistical analyses (Mann–Whitney U test, Kruskal–Wallis test, Spearman correlation, Benjamini–Hochberg correction and principal component analysis (PCA)) were performed using R and GraphPad Prism 5.01 software (GraphPad Software, USA). When testing the subgroups, a prior heterogeneity test for differences between subgroups was performed.

## Results

### Serum inflammatory pattern associated with a high cumulative GC dose

**Figure 1:**
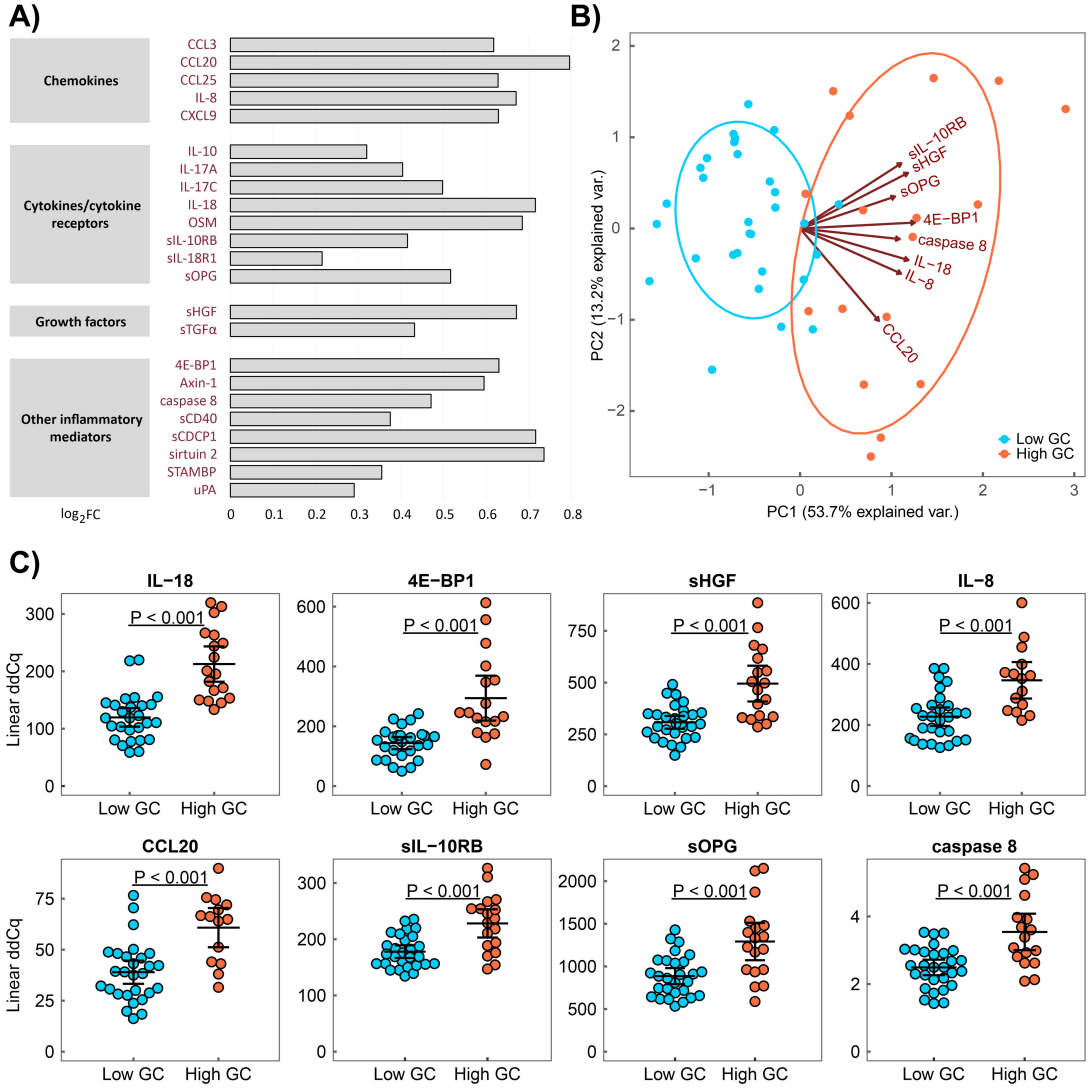
Serum proteins associated with a high cumulative GC dose in active RA. **(A)** Serum protein pattern associated with a high cumulative GC dose presented as a log2 fold change (FC) of group medians compared with a low GC dose. **(B)** Principal component analysis (PCA) of patients with low (blue) and high (red) cumulative GC doses characterised by a combination of eight serum protein levels (IL–18, 4E–BP1, sHGF, IL–8, CCL20, sIL–10RB, sOPG and caspase 8). **(C)** Distribution of protein serum levels that differ between patients with RA with a high cumulative GC dose (High GC) and those with a low cumulative GC dose (Low GC) with active RA, as identified through PCA. Group means are indicated by horizontal bars; error bars indicate 95% CI.

To determine the impact of a cumulative GC dose on the serum levels of proteins associated with inflammation in patients with RA, we compared serum protein levels between patients with low and high cumulative GC doses. Patient groups were formed based on the total cumulative GC dose, calculated as the sum of oral or intravenous GC doses during all the treatment periods, with a cut-off value of 20 g (low/high, n = 49/23) based on the mean of the values in the patient cohort. Because disease activity, according to the prior heterogeneity test, also influences serum protein levels, we compared patients with low and high GC doses separately within active and non-active RA subgroups. Patients with a high cumulative GC dose within the active RA group had elevated serum levels in 23 inflammation-related proteins compared with patients with a low dose (chemokines: CCL3, CCL20, CCL25, IL–8 and CXCL9; cytokines/cytokine receptors: IL–10, IL–17A, IL–17C, IL–18, OSM, sIL–10RB, sIL–18R1 and sOPG; growth factors: sTGFα and sHGF; other inflammatory mediators: caspase 8, STAMBP, sCDCP1, sirtuin 2, 4E–BP1, sCD40, uPA and axin–1; p_corr_ < 0.05; **Figure 1**, **Supplementary Figure S1**, **Supplementary Table S2**). Of these, the levels of nine proteins (IL–8, CCL20, IL–17A, IL–17C, IL–18, 4E–BP1, caspase 8, sCD40 and sOPG) correlated with a cumulative GC dose (r ≥ 0.40, p ≤ 0.01, **Supplementary Figure S2**) in active RA. An upward trend in serum IL–6 level was observed in patients with high cumulative GC dose within the active RA group when compared to the low GC dose group (p_corr_ = 0.07). In addition, the PCA revealed that the serum levels of eight proteins (IL–18, 4E–BP1, sHGF, IL–8, CCL20, sIL–10RB, sOPG and caspase 8; **Figure 1**) can distinguish patients in the high and low cumulative GC dose groups within the active RA group. In the non-active RA group, no differences in the analysed serum protein levels were identified between the high and low GC dose groups (p_corr_ > 0.05, **Supplementary Table S3**).

Next, we performed correlation and regression analyses and subanalyses in patient groups to assess the relationship between the clinical parameters. Among the investigated demographic and clinical parameters (age, gender, disease duration, GC doses, HAQ score, laboratory markers, etc.), only disease duration and cumulative GC dose were found to be associated (**Supplementary Table S4**, **Supplementary Figure S3**). Notably, when comparing serum protein levels between patients with active RA with low and high cumulative GC doses using a lower cut-off value of 15 g (low/high, n = 23/25), which is the median of the values in the patient cohort, no difference was found after correction for multiple testing in the analysed serum protein levels, except in CCL20 (p_corr_ = 0.03, **Supplementary Table S5**).

When the active RA subgroups were stratified by DAS28, most proteins exhibited the same serum inflammatory pattern associated with a high cumulative GC dose as in case of subgroups based on SDAI (p < 0.05, **Supplementary Table S6**), except for IL-10 (p = 0.06), CCL3 (p = 0.07) and IL-17C (p = 0.08), in which an upwards trend was observed. Additionally, TGF-β1 and sLIFR were found to be associated with a high cumulative GC (p_corr_ > 0.05), and these proteins were also on the significance borderline when tested based on SDAI (p = 0.05 and p = 0.15, respectively) (**Supplementary Table S2**).

To assess the serum protein pattern associated with RA irrespective of the GC dose, we compared the serum protein levels in patients with RA and healthy controls. Of the analysed serum proteins, 28 were upregulated and 3 (IL–7, FGF19 and CST5) were downregulated in RA (p_corr_ ≤ 0.05; **Supplementary Table S7**, **Supplementary Figure S4**). The upregulated proteins were cytokines/soluble receptors (CCL3, CCL4, CCL7, CXCL1, IL–8/CXCL8, CXCL9, CXCL10, CXCL11, IL–6, IL–10, sIL–18R1, OSM, sTNFSF14, sTNFRSF9, sTRAIL and sTRANCE), growth factors (FGF23, sHGF and sTGFα) and other inflammatory mediators (axin–1, caspase 8, CST5, EN–RAGE, MMP1, sCD40, sirtuin 2, sSLAMF1, STAMBP and SULT1A1).

### Clinical characteristics associated with a high GC dose

**Figure 2:**
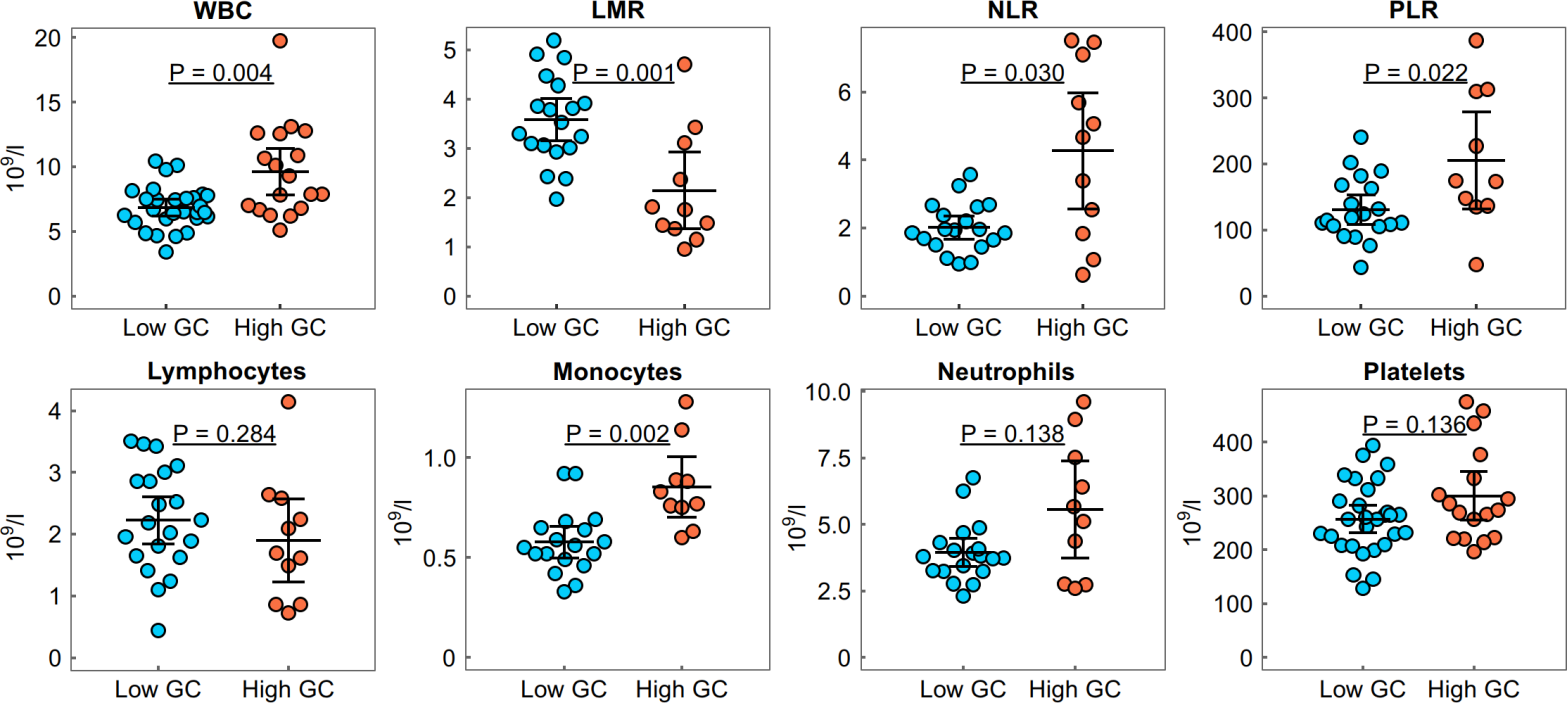
Distribution of the white blood cell count (WBC), neutrophil–lymphocyte ratio (NLR), platelet–lymphocyte ratio (PLR) and lymphocyte–monocyte ratio (LMR) in the high and low GC dose groups with active RA. Group means are indicated by horizontal bars; error bars indicate 95% CI.

Next, we compared the clinical and laboratory characteristics of patients with low and high cumulative GC doses, as well as in subgroups according to disease activity. In the active RA group, an increased white blood cell (WBC) count, an increased neutrophil–lymphocyte ratio (NLR) and platelet–lymphocyte ratio (PLR) and a decreased lymphocyte–monocyte ratio (LMR) were identified in the high GC dose group compared with the low dose group (p < 0.05, **Figure 2**). No differences in the levels of rheumatoid factor (RF), anti-citrullinated protein antibodies (ACPA) or C-reactive protein (CRP), or in the erythrocyte sedimentation rate (ESR), were found between the high and low GC dose groups (p > 0.05, **Table 1**). Our study also identified an increased proportion of patients with osteoporosis in the high cumulative GC dose group compared with the low dose group (78% vs 37%, p = 0.001) and a correlation between the cumulative GC dose and the functional disability of patients, as assessed by the HAQ score (r = 0.60, p < 0.0001). In the non-active RA group, no differences in the analysed clinical characteristics were found between the high and low GC dose groups (p > 0.05).

## Discussion

Although GCs have been used in the treatment of RA for more than 70 years, there is limited knowledge of how the long-term administration of higher doses of GCs affects the systemic inflammatory response. In a heavily pre-treated real-world cohort with a median disease duration of 14 years, we revealed that a high cumulative GC dose is associated with increased serum levels in inflammation-related mediators and deregulated blood counts in active RA.

GCs exert both anti-inflammatory and immunosuppressive effects in RA through several mechanisms, including immune cell polarisation and activation and the secretion of cytokines, enzymes and collagenase (3). In this study, we evaluated the effect of a high cumulative GC dose on the serum levels of inflammatory mediators and blood counts in a real-world cohort of patients with RA receiving long-term treatment. The major effect was detected in patients with high cumulative GC doses of 20 g or more, which was not observed in subgroups below GC doses of 15 g or in non-active RA. In active RA, we found a total of 23 proteins associated with inflammation to be upregulated in the sera of patients with a high cumulative GC dose when compared with the low GC dose group. The top upregulated proteins in the high GC dose group with active RA were cytokines/soluble cytokine receptors IL–8, CCL20, IL–18, sIL–10RB and sOPG and inflammatory-related proteins 4E–BP1, sHGF and caspase 8. All of these upregulated proteins have previously been reported in RA (IL–8 (9), CCL20 (9), IL–18 (10), IL–10RB (11), OPG (12), 4E–BP1 (13), HGF (14) and caspase 8 (15)); however, these proteins were reported in relationship with GC therapy in other pathologies/conditions, but not in RA. The induction of chemokines CCL20 and IL–8/CXCL8 by GCs was previously reported in human macrophages (16), enhanced CCL20 expression was reported in GC-insensitive neutrophilic airway inflammation in asthma (17) and elevated CCL20 expression was found in keratinocytes in GC-exacerbated skin conditions (18). Moreover, IL–18 was reported to be stimulated in the adrenal cortex by adrenocorticotropic hormone treatment and not inhibited by the direct action of GCs (19). Regarding OPG, a decoy receptor of RANKL involved in the regulation of bone resorption, discordant observations were published, with studies reporting elevated OPG expression following GC treatment in a mouse model of GC-induced osteonecrosis (20) as well as reduced OPG expression after GC therapy (21, 22). Regarding 4E–BP1, a member of a family of translation repressor proteins, its dephosphorylation was reported in rats after GC treatment (23). Moreover, the deletion of a 4E–BP1 gene is a proposed mechanism for GC resistance in the Raji cell line, established in a patient with Burkitt lymphoma (24). In line with our results, the increased serum levels of HGF, a growth factor involved in bone metabolism, were also demonstrated in patients with ankylosing spondylitis treated with GCs compared with those without GC therapy (25). Regarding caspase 8, the induction of the apoptosis of thymocytes by GCs through the activation of caspase 8 was reported (26). Although the altered serum levels of IL-10 and its receptor IL-10RB in RA have been described by us and others (11), IL-10RB has not yet been associated with GC treatment.

The difference in the protein pattern observed in the high GC group might also indicate that patients with RA may benefit from new therapies targeting the molecules identified in this study. Among them is an anti-CD40 monoclonal antibody, abiprubart, which has recently been shown to reduce disease activity in patients with refractory RA (27). Anti-CCL3 monoclonal antibodies strongly inhibited joint injury and bone erosion in a collagen-induced arthritis (CIA) mouse model (28); AMG487, the antagonist of CXCR3, a receptor for chemokines CXCL9–11, also supressed RA in a CIA model (29). Regarding the IL-17A blockade, clinical trials have shown mostly modest effects in RA, especially compared with the clinical efficacy observed in psoriatic arthritis, psoriasis and spondyloarthritis (30). Similarly, a humanised anti-OSM did not exhibit efficacy in a RA clinical trial (31). Nevertheless, the effect of IL-17A or OSM blockade in specific groups of patients with RA, such as those with high levels of IL-17A or OSM in serum or synovial fluid or those with rapidly progressing disease, may be of a high interest. Testing drugs in combinations, such as the dual cytokine blockade of IL-17A/TNF in stratified patient groups, may be an additional avenue to pursue (30). Although these approaches may not add more value than current treatment options for most patients with RA, they may be beneficial for those patients for whom current treatments are not sufficient.

It should also be noted that patients in the high GC group may represent a specific subgroup of patients with a severe disease course who often require the intensification of treatment, including GC therapy, to reduce disease activity. In all the patients, the disease management was compliant with current recommendations and was guided by efforts to reduce or discontinue GC treatment whenever a treatment response was achieved (5). Whether the elevated levels of the detected proteins are related to a specific RA phenotype or to the GC dose, as supported by observations in other pathologies, deserves further investigation. However, these patients may benefit from new therapeutic approaches, such as the targeting of molecules identified in this study.

When considering the clinical and laboratory characteristics associated with a high cumulative GC dose, we did not observe any association with classic RA laboratory markers (ACPA, RF, ESR and CRP), but we identified an increased WBC count, increased NLR and PLR and decreased LMR compared with the low GC dose group. This observation is in line with a previous study reporting GC-induced leucocytosis, attributed predominantly to a rise in neutrophils that coincided with monocytosis and a variable degree of lymphopenia in several autoimmune diseases (32). Furthermore, a decreased LMR and increased NLR and PLR have previously been associated with RA disease activity and a poorer treatment response (33, 34). A recent study in genetically modified RA mouse models revealed that GCs may exert different effects on diverse cells in the joint surroundings, such as anti-inflammatory effects on macrophages, mast cells and chondrocytes and pro-inflammatory effects on fibroblast-like synoviocytes, myocytes, osteoblasts and osteocytes (35). Our study also confirmed an increased proportion of patients with osteoporosis in the high cumulative GC dose group compared with the low GC group.

In addition, our protein study revealed that approximately one third of proteins that were found to be associated with RA compared with healthy participants were not influenced by GCs. Among the deregulated proteins associated with RA, the upregulation of STAMBP and SULT1A1 and downregulation of FGF19 were detected; these proteins have not been previously described in RA. The STAMBP protein is an important regulator of innate immunity through the direct modulation of the NLRP3 inflammasome and IL–1β secretion; STAMBP inhibition impairs the TLR-mediated increase in NLRP7 expression levels and dampens IL–1β release (36, 37). Thus, elevated STAMBP levels might further contribute to the pro-inflammatory milieu of RA. Regarding SULT1A1, an enzyme catalysing the sulfonation of many phenolic molecules, including endogenous compounds (e.g. estradiol and iodothyronines), environmental xenobiotics and drugs (38), its downregulation in the plasma of patients with inactive systemic juvenile idiopathic arthritis (39) and upregulation in the sera of patients with ulcerative colitis have been reported (40). Regarding FGF19, an endocrine hormone regulating various metabolic processes, its decreased level was reported in the sera of patients with overt hypothyroidism and subclinical hypothyroidism (41); however, its role in autoimmunity is unclear.

The limitation of this study lies in the modest sample size, which does not allow for more subgroups to be considered to fully explore the heterogeneity of RA. Given the complexity of the clinical data and the heterogeneity of RA, further analysis should be performed to reveal potential associations with clinical parameters and control for potential confounding factors. However, this study in a unique cohort of patients receiving long-term treatment has identified biomarkers for future studies in larger cohorts.

In conclusion, this is the first study to report the increased serum levels of inflammatory proteins and deregulated blood counts in active RA associated with a high cumulative dose of GCs, further contributing to the understanding of the long-term impact of GC treatment in RA. This study highlights the importance of improving care for patients with high cumulative GC doses, as increased systemic inflammation may affect their treatment response.

## Data Availability

The raw data supporting the conclusions of this article will be made available by the authors, without undue reservation.
