# Supplementary material for: High cumulative glucocorticoid dose is associated with increased levels of inflammation-related mediators in active rheumatoid arthritis
Source: Front Immunol. 2024 Dec 18;15:1505615. doi: 10.3389/fimmu.2024.1505615 (PMC11688219; doi:10.3389/fimmu.2024.1505615)
Supplement: Supplementary file 1 [file Table1.docx]

The Supplementary File

**High cumulative glucocorticoid dose is associated with increased levels of inflammation-related mediators in active rheumatoid arthritis**

Anna Petrackova et al.

**Contents:**

Table S1: List of investigated proteins.

Table S2: Serum levels of studied proteins in patients with low and high GC dose within active RA group.

Table S3: Serum levels of studied proteins in patients with low and high GC dose within non-active RA group.

Table S4: Regression analysis between cumulative glucocorticoid (GC) dose and other continuous clinical parameters.

Table S5: Serum protein levels in active RA patients with low and high cumulative GC dose using a lower cut-off value of 15 g.

Table S6: Serum levels of studied proteins in patients with low and high GC dose within active RA group when using DAS28.

Table S7: Serum levels of studied proteins in patients with RA when compared to control group of healthy individuals.

Figure S1: Distribution of serum levels of proteins different between RA patients with high cumulative GC dose (High GC) when compared to the low cumulative GC dose group (Low GC) within active RA. This figure does not show the highest ranked proteins identified in PCA, which are shown in Figure 1.

Figure S2: Correlation of serum protein levels with cumulative GC dose within active RA.

Figure S3: Correlation matrix of clinical parameters of patients with RA.

Figure S4: Serum proteins associated with RA. Deregulated serum proteins associated with RA when compared to control group of healthy individuals, presented as log2 fold change (FC) of group medians.

**Table S1: List of investigated proteins.**

| Symbol | Protein name | Symbol | Protein name |
| --- | --- | --- | --- |
| 4E-BP1 | Eukaryotic translation initiation factor 4E-binding protein 1 | sCD40 | CD40L receptor, soluble |
| AXIN1 | Axin 1 | sCD244 | Natural killer cell receptor 2B4, soluble |
| BDNF | Brain-derived neurotrophic factor | sCDCP1 | CUB domain-containing protein 1, soluble |
| bNGF | Beta-nerve growth factor | sCX3CL1 | Fractalkine, soluble |
| CASP8 | Caspase 8 | sDNER | Delta and Notch-like epidermal growth factor-related receptor, soluble |
| CCL2 | Monocyte chemotactic protein 1 | sFlt3L | Fms-related tyrosine kinase 3 ligand, soluble |
| CCL3 | C-C motif chemokine 3 | sHGF | Hepatocyte growth factor |
| CCL4 | C-C motif chemokine 4 | sIL-10RB | Interleukin-10 receptor subunit beta, soluble |
| CCL7 | Monocyte chemotactic protein 3 | sIL-15RA | Interleukin-15 receptor subunit alpha, soluble |
| CCL8 | Monocyte chemotactic protein 2 | sIL-18R1 | Interleukin-18 receptor 1, soluble |
| CCL11 | Eotaxin-1 | Sirtuin 2 | SIR2-like protein 2 |
| CCL13 | Monocyte chemotactic protein 4 | sLIFR | Leukemia inhibitory factor receptor, soluble |
| CCL19 | C-C motif chemokine 19 | sOPG | Osteoprotegerin, soluble |
| CCL20 | C-C motif chemokine 20 | sPDL1 | Programmed cell death 1 ligand 1, soluble |
| CCL23 | C-C motif chemokine 23 | sSCF | Stem cell factor, soluble |
| CCL25 | C-C motif chemokine 25 | sSLAMF1 | Signaling lymphocytic activation molecule, soluble |
| CCL28 | C-C motif chemokine 28 | SULT1A1 | Sulfotransferase 1A1 |
| CSF1 | Macrophage colony-stimulating factor 1 | STAMBP | STAM-binding protein |
| CST5 | Cystatin D | sTGFα | Transforming growth factor alpha, soluble |
| CXCL1 | C-X-C motif chemokine 1 | sTNFβ | TNF-beta, soluble |
| CXCL5 | C-X-C motif chemokine 5 | sTNFRSF9 | Tumor necrosis factor receptor superfamily member 9, soluble |
| CXCL6 | C-X-C motif chemokine 6 | sTNFSF14 | Tumor necrosis factor ligand superfamily member 14, soluble |
| CXCL9 | C-X-C motif chemokine 9 | sTRAIL | TNF-related apoptosis-inducing ligand, soluble |
| CXCL10 | C-X-C motif chemokine 10 | sTRANCE | TNF-related activation-induced cytokine, soluble |
| CXCL11 | C-X-C motif chemokine 11 | sTWEAK | Tumor necrosis factor (Ligand) superfamily, member 12 |
| EN.RAGE | Protein S100-A12 | uPA | Urokinase-type plasminogen activator |
| FGF5 | Fibroblast growth factor 5 | VEGFA | Vascular endothelial growth factor A |
| FGF19 | Fibroblast growth factor 19 | Analytes below the limit of detection | |
| FGF21 | Fibroblast growth factor 21 | sIL-2RB | Interleukin-2 receptor subunit beta, soluble |
| FGF23 | Fibroblast growth factor 23 | sIL-10RA | Interleukin-10 receptor subunit alpha, soluble |
| GDNF | Glial cell line-derived neurotrophic factor | sIL-20RA | Interleukin-20 receptor subunit alpha, soluble |
| IL-6 | Interleukin-6 | sIL-22RA1 | Interleukin-22 receptor subunit alpha-1, soluble |
| IL-7 | Interleukin-7 | TSLP | Thymic stromal lymphopoietin |
| IL-8 | Interleukin-8 | TNFα | Tumor necrosis factor alpha |
| IL-10 | Interleukin-10 | ARTN | Artemin |
| IL-12β | Interleukin-12 subunit beta | IFNγ | Interferon gamma |
| IL-17A | Interleukin-17A | IL-1α | Interleukin-1 alpha |
| IL-17C | Interleukin-17C | IL-2 | Interleukin-2 |
| IL-18 | Interleukin-18 | IL-4 | Interleukin-4 |
| LAP-TGFβ1 | Latency-associated peptide transforming growth factor beta-1 | IL-5 | Interleukin-5 |
| MMP1 | Matrix metalloproteinase-1 | IL-13 | Interleukin-13 |
| MMP10 | Matrix metalloproteinase-10 | IL-20 | Interleukin-20 |
| NT3 | Neurotrophin-3 | IL-24 | Interleukin-24 |
| OSM | Oncostatin-M | IL-33 | Interleukin-33 |
| sADA | Adenosine deaminase, soluble | LIF | Leukemia inhibitory factor |
| sCD5 | T-cell surface glycoprotein CD5, soluble | NRTN | Neurturin |
| sCD6 | T cell surface glycoprotein CD6 isoform, soluble |  |  |

**Table S2: Serum levels of studied proteins in patients with low and high GC dose within active RA group.**

|  |  |  |  | Low GC dose | | | High GC dose | | |
| --- | --- | --- | --- | --- | --- | --- | --- | --- | --- |
| Analyte | p value | Benjamini-Hochberg Correction | Fold Change | Mean | CI low | CI high | Mean | CI low | CI high |
| IL-18 | 2.55779E-06 | 7.07655E-05 | 0.67033633 | 119.6747494 | 103.5367612 | 135.8127376 | 212.6932052 | 181.9129993 | 243.4734111 |
| 4E-BP1 | 2.20607E-05 | 0.000366208 | 0.602139755 | 143.6876132 | 123.5325005 | 163.842726 | 294.0691335 | 218.9372511 | 369.2010158 |
| sHGF | 0.000152821 | 0.002114023 | 0.592028608 | 308.2122904 | 277.2770997 | 339.1474811 | 495.122592 | 408.6730966 | 581.5720875 |
| IL-8 | 0.000527248 | 0.005616891 | 0.552928424 | 227.3027851 | 197.1338973 | 257.4716728 | 346.4619272 | 286.7540938 | 406.1697605 |
| CCL20 | 0.000576967 | 0.005616891 | 0.693490625 | 38.97717546 | 33.28634136 | 44.66800956 | 60.81751826 | 51.20264883 | 70.4323877 |
| sIL-10RB | 0.000621192 | 0.005616891 | 0.361387693 | 177.8813258 | 166.6505043 | 189.1121473 | 228.1545951 | 203.0460783 | 253.2631119 |
| sOPG | 0.000676734 | 0.005616891 | 0.465211247 | 886.0414435 | 792.1830746 | 979.8998123 | 1291.986307 | 1072.45243 | 1511.520184 |
| caspase 8 | 0.000820397 | 0.006190269 | 0.356604327 | 2.491586312 | 2.26170692 | 2.721465703 | 3.541854503 | 3.001567341 | 4.082141665 |
| STAMBP | 0.000899668 | 0.006222705 | 0.34245159 | 10.93283923 | 9.564576459 | 12.30110199 | 15.6553752 | 13.10955464 | 18.20119576 |
| sCD40 | 0.001501301 | 0.009539442 | 0.301373117 | 885.8325217 | 807.6366071 | 964.0284362 | 1149.169307 | 1014.754301 | 1283.584313 |
| IL-17C | 0.001609062 | 0.009539442 | 0.301466904 | 1.810166737 | 1.655509285 | 1.964824188 | 2.755724354 | 2.116173481 | 3.395275227 |
| OSM | 0.002617929 | 0.013790038 | 0.652883354 | 35.29379359 | 29.27024438 | 41.31734281 | 53.75323854 | 44.06581495 | 63.44066212 |
| sCDCP1 | 0.002658321 | 0.013790038 | 0.547750868 | 7.891885845 | 6.995281379 | 8.788490311 | 14.18569604 | 9.917525924 | 18.45386616 |
| uPA | 0.002959021 | 0.014446983 | 0.248330549 | 813.1882918 | 749.5539713 | 876.8226123 | 989.6631471 | 905.3710099 | 1073.955284 |
| CCL3 | 0.005146419 | 0.023730711 | 0.448933453 | 6.931525686 | 5.849799027 | 8.013252344 | 9.742049367 | 8.035670852 | 11.44842788 |
| sTGFα | 0.005432481 | 0.023731363 | 0.34881973 | 12.53633677 | 10.63356388 | 14.43910967 | 20.74478957 | 15.74712175 | 25.74245739 |
| IL-10 | 0.007856144 | 0.031623106 | 0.33329067 | 13.77194394 | 11.98640851 | 15.55747937 | 19.22287249 | 15.04904016 | 23.39670482 |
| axin-1 | 0.008001027 | 0.031623106 | 0.536875181 | 2.289096227 | 1.964602884 | 2.613589569 | 3.312622079 | 2.616845775 | 4.008398383 |
| sirtuin 2 | 0.010409582 | 0.039272514 | 0.687135082 | 17.67893626 | 13.93686128 | 21.42101124 | 26.02081764 | 20.60849047 | 31.43314482 |
| IL-17A | 0.012433387 | 0.044868311 | 0.375574862 | 1.084513776 | 1.011981745 | 1.157045808 | 1.310421194 | 1.163956754 | 1.456885634 |
| CCL25 | 0.013808411 | 0.045843926 | 0.594719451 | 81.64206404 | 69.86403616 | 93.42009192 | 135.3229502 | 99.86548808 | 170.7804123 |
| CXCL9 | 0.015065786 | 0.046990394 | 0.527579332 | 247.9489748 | 210.6814059 | 285.2165438 | 392.4530124 | 274.2904437 | 510.6155811 |
| sIL-18R1 | 0.015286032 | 0.046990394 | 0.164733586 | 215.1124298 | 193.3159405 | 236.908919 | 245.1211911 | 233.8434966 | 256.3988857 |
| sFlt3L | 0.018656747 | 0.055303929 | 0.287889364 | 475.8447938 | 422.5879098 | 529.1016779 | 624.419337 | 513.7496924 | 735.0889816 |
| IL-6 | 0.023028954 | 0.064888353 | 1.917602624 | 28.5570389 | 19.07559678 | 38.03848102 | 114.8950021 | 47.38810355 | 182.4019007 |
| FGF5 | 0.023536153 | 0.064888353 | 0.156688184 | 2.345019412 | 2.167030582 | 2.523008243 | 2.792662999 | 2.405212685 | 3.180113312 |
| sTNFSF14 | 0.024235409 | 0.064888353 | 0.257013375 | 49.37984247 | 42.52898588 | 56.23069906 | 63.29868785 | 53.98616125 | 72.61121445 |
| CCL11 | 0.02782362 | 0.072167514 | 0.189235683 | 355.77107 | 321.1686317 | 390.3735083 | 437.09423 | 368.2882512 | 505.9002087 |
| FGF21 | 0.029896857 | 0.075195126 | 1.387173176 | 23.20480052 | 15.56048553 | 30.84911551 | 44.69285157 | 26.89807001 | 62.48763314 |
| sPDL1 | 0.038358295 | 0.088437181 | 0.181049403 | 28.1815967 | 25.67880897 | 30.68438442 | 33.03591712 | 29.29510151 | 36.77673272 |
| CSF1 | 0.041909297 | 0.094012746 | 0.071464576 | 283.5868635 | 263.7194304 | 303.4542966 | 316.1605335 | 293.6094984 | 338.7115685 |
| MMP10 | 0.044106461 | 0.096337796 | 0.176715931 | 521.2514608 | 433.6406316 | 608.8622901 | 610.8760336 | 519.3014852 | 702.4505821 |
| sIL-15RA | 0.04618572 | 0.096373172 | 0.148698355 | 1.715560831 | 1.604719786 | 1.826401876 | 1.90650469 | 1.747523615 | 2.065485766 |
| SULT1A1 | 0.046444902 | 0.096373172 | 1.008449711 | 13.66934111 | 9.356291173 | 17.98239104 | 20.00372256 | 13.54574576 | 26.46169935 |
| GDNF | 0.050812199 | 0.102863719 | 0.132883885 | 3.448313369 | 3.2041416 | 3.692485139 | 4.209132304 | 3.523333495 | 4.894931114 |
| LAP-TGFB1 | 0.054015316 | 0.106744554 | 0.328685814 | 172.1633397 | 152.8233726 | 191.5033067 | 198.1262894 | 177.7853081 | 218.4672706 |
| CCL23 | 0.058314064 | 0.111071679 | 0.292849594 | 638.519755 | 580.356527 | 696.682983 | 780.5483938 | 659.3517654 | 901.7450221 |
| CXCL10 | 0.058881372 | 0.111071679 | 0.484523571 | 669.0991066 | 546.1473185 | 792.0508947 | 1011.97851 | 717.9124507 | 1306.044569 |
| MMP1 | 0.060560383 | 0.111700261 | 0.681954438 | 1155.405704 | 880.4692279 | 1430.34218 | 1537.186178 | 1212.518911 | 1861.853446 |
| sSLAMF1 | 0.063572387 | 0.114706698 | 0.2397077 | 7.891282484 | 6.883659576 | 8.898905392 | 10.31189446 | 7.908329703 | 12.71545922 |
| bNGF | 0.065206102 | 0.115151202 | 0.101905116 | 2.786262291 | 2.590562434 | 2.981962148 | 3.154085833 | 2.822238694 | 3.485932972 |
| sCX3CL1 | 0.067743038 | 0.117139003 | 0.010445378 | 77.40643516 | 70.81637923 | 83.99649109 | 90.59083167 | 81.38169098 | 99.79997235 |
| VEGFA | 0.07260593 | 0.120525844 | 0.137643181 | 1715.600555 | 1474.091778 | 1957.109332 | 2194.016992 | 1775.495374 | 2612.53861 |
| CCL7 | 0.089242269 | 0.145237419 | 0.117287138 | 10.50185498 | 7.995347665 | 13.0083623 | 16.20705125 | 9.945429382 | 22.46867312 |
| CXCL1 | 0.111104681 | 0.177340164 | 0.109569472 | 363.6941193 | 330.4556596 | 396.932579 | 424.5287646 | 365.1009515 | 483.9565777 |
| CXCL11 | 0.113691042 | 0.178044462 | 0.094293701 | 154.8419158 | 134.0161473 | 175.6676844 | 205.9933033 | 159.1625548 | 252.8240519 |
| CCL4 | 0.126502829 | 0.194439534 | 0.109542818 | 81.22223317 | 71.40625243 | 91.03821391 | 103.5731635 | 79.0697701 | 128.0765568 |
| EN-RAGE | 0.131921505 | 0.199081544 | 0.394743666 | 63.11627833 | 51.34585385 | 74.8867028 | 87.53642923 | 60.89195103 | 114.1809074 |
| CCL28 | 0.15048414 | 0.222949564 | 0.086526049 | 2.186511374 | 1.953214175 | 2.419808574 | 2.499277145 | 2.183587076 | 2.814967215 |
| sLIFR | 0.153109942 | 0.222949564 | 0.071773463 | 9.5685197 | 8.813831678 | 10.32320772 | 10.22958865 | 9.358797682 | 11.10037961 |
| FGF23 | 0.164926312 | 0.236015239 | 0.125490943 | 3.824199682 | 3.503157716 | 4.145241648 | 4.238663988 | 3.669122037 | 4.808205939 |
| sADA | 0.191622304 | 0.26957036 | 0.129025427 | 25.04233033 | 22.85305949 | 27.23160117 | 27.5977701 | 24.72543968 | 30.47010051 |
| sCD244 | 0.203903346 | 0.282066295 | 0.1329111 | 64.95544617 | 58.48910274 | 71.4217896 | 68.18497949 | 62.78179783 | 73.58816115 |
| sTWEAK | 0.228816346 | 0.311340275 | 0.12212908 | 336.4694991 | 309.7838464 | 363.1551518 | 363.8929141 | 313.6384919 | 414.1473364 |
| CCL2 | 0.245594331 | 0.328779507 | 0.086734863 | 1205.815089 | 1061.129939 | 1350.500239 | 1412.542843 | 1151.98713 | 1673.098556 |
| sTNFRSF9 | 0.342973029 | 0.444793147 | 0.094293701 | 97.01330819 | 85.96656949 | 108.0600469 | 107.1279223 | 88.42927125 | 125.8265733 |
| CCL19 | 0.401670167 | 0.512901906 | -0.117297004 | 621.8670115 | 503.5891124 | 740.1449106 | 508.2247019 | 414.8209344 | 601.6284694 |
| sTNFβ | 0.419134429 | 0.527093297 | -0.09575639 | 10.35887681 | 9.0281378 | 11.68961582 | 9.401374652 | 7.765085325 | 11.03766398 |
| CST5 | 0.48166152 | 0.587077987 | 0.101931587 | 100.5635094 | 86.47683188 | 114.650187 | 98.91900171 | 88.57342974 | 109.2645737 |
| IL-12B | 0.484476213 | 0.587077987 | 0.366040257 | 19.70007872 | 16.23781575 | 23.16234169 | 21.46981888 | 16.9319195 | 26.00771825 |
| CCL8 | 0.488052784 | 0.587077987 | 0.021012126 | 1100.154469 | 950.9232569 | 1249.385681 | 1161.697156 | 1020.294454 | 1303.099858 |
| sTRAIL | 0.540945169 | 0.641406415 | -0.070167137 | 257.4003033 | 232.5991632 | 282.2014434 | 257.7264029 | 236.5639976 | 278.8888083 |
| CCL13 | 0.566263117 | 0.66196956 | -0.00348966 | 10.92061044 | 9.590420853 | 12.25080002 | 9.896678359 | 8.350634041 | 11.44272268 |
| FGF19 | 0.632647545 | 0.729302032 | -0.098749537 | 184.0386373 | 143.4526542 | 224.6246203 | 190.571137 | 144.5734834 | 236.5687907 |
| sCD6 | 0.707276417 | 0.798632246 | -0.013767296 | 9.927893072 | 8.766115797 | 11.08967035 | 10.90500081 | 8.825355977 | 12.98464564 |
| sDNER | 0.712033569 | 0.798632246 | -0.030692719 | 157.5016905 | 147.7470548 | 167.2563262 | 154.0406179 | 144.1533591 | 163.9278766 |
| CXCL5 | 0.756135336 | 0.836789771 | 0.009960103 | 5270.97479 | 4673.38412 | 5868.565461 | 5148.126593 | 4290.810046 | 6005.44314 |
| sSCF | 0.791065371 | 0.863697873 | -0.057356998 | 499.4283476 | 442.5894235 | 556.2672716 | 507.9929801 | 449.9832718 | 566.0026884 |
| sTRANCE | 0.801261882 | 0.863697873 | -0.159103585 | 30.81512645 | 25.53161748 | 36.09863542 | 29.71703776 | 21.65914601 | 37.7749295 |
| NT3 | 0.828790443 | 0.878381041 | 0.01395948 | 2.63140742 | 2.389565634 | 2.873249206 | 2.584063623 | 2.274841677 | 2.893285569 |
| BDNF | 0.836049425 | 0.878381041 | 1.329467173 | 9.826627279 | 3.453860977 | 16.19939358 | 8.668132295 | 2.360293334 | 14.97597126 |
| IL-7 | 0.856324924 | 0.888437109 | -0.012606163 | 30.61347379 | 26.99051486 | 34.23643272 | 31.7629597 | 25.97827018 | 37.54764923 |
| sCD5 | 0.895511878 | 0.906432755 | -0.013743603 | 12.21166432 | 11.11842355 | 13.30490509 | 12.84174554 | 10.63243093 | 15.05106016 |
| CXCL6 | 0.938223798 | 0.938223798 | 0.042240416 | 437.0903168 | 376.2653295 | 497.9153041 | 422.3991793 | 359.414041 | 485.3843175 |

Legend: CI: 95% confidence interval

**Table S3: Serum levels of studied proteins in patients with low and high GC dose within non-active RA group.**

|  |  |  |  | Low GC dose | | | High GC dose | | |
| --- | --- | --- | --- | --- | --- | --- | --- | --- | --- |
| Analyte | p value | Benjamini-Hochberg Correction | Fold Change | Mean | CI low | CI high | Mean | CI low | CI high |
| BDNF | 0.002 | 0.08 | 318.025 | 41.5 | 4.982 | 78.018 | 539.632 | 135.728 | 943.535 |
| sFlt3L | 0.008 | 0.141 | 0.516 | 362.536 | 312.853 | 412.22 | 531.938 | 443.664 | 620.213 |
| sLIFR | 0.009 | 0.141 | 0.189 | 8.956 | 8.368 | 9.544 | 11.153 | 9.584 | 12.722 |
| MMP1 | 0.011 | 0.148 | -0.641 | 1261.269 | 995.924 | 1526.614 | 433.151 | 266.504 | 599.798 |
| CCL25 | 0.024 | 0.162 | 0.429 | 68.099 | 57.266 | 78.932 | 94.675 | 68.2 | 121.15 |
| FGF21 | 0.019 | 0.162 | 1.789 | 13.208 | 8.226 | 18.189 | 35.338 | 6.532 | 64.143 |
| sCX3CL1 | 0.025 | 0.162 | 0.444 | 84.168 | 73.253 | 95.082 | 111.802 | 84.96 | 138.644 |
| sDNER | 0.026 | 0.162 | 0.137 | 145.615 | 134.025 | 157.204 | 171.592 | 164.753 | 178.431 |
| SULT1A1 | 0.027 | 0.162 | -0.608 | 20.417 | 13.262 | 27.572 | 6.564 | 3.044 | 10.083 |
| sTNFSF14 | 0.015 | 0.162 | -0.283 | 52.089 | 45.92 | 58.258 | 37.825 | 27.009 | 48.642 |
| EN-RAGE | 0.044 | 0.242 | 0.608 | 32.476 | 23.28 | 41.673 | 49.129 | 34.798 | 63.46 |
| axin-1 | 0.057 | 0.297 | -0.325 | 3.058 | 2.451 | 3.664 | 1.995 | 1.423 | 2.567 |
| CXCL6 | 0.087 | 0.376 | 0.24 | 369.538 | 310.995 | 428.08 | 478.927 | 447.928 | 509.927 |
| sPDL1 | 0.081 | 0.376 | 0.181 | 27.703 | 24.782 | 30.624 | 34.187 | 25.524 | 42.851 |
| sTNFRSF9 | 0.086 | 0.376 | 0.5 | 92.059 | 78.081 | 106.036 | 133.681 | 76.285 | 191.077 |
| sTRAIL | 0.102 | 0.402 | 0.157 | 228.707 | 201.772 | 255.642 | 275.076 | 230.344 | 319.807 |
| bNGF | 0.179 | 0.474 | 0.053 | 2.771 | 2.632 | 2.91 | 2.942 | 2.765 | 3.12 |
| CCL11 | 0.151 | 0.474 | 0.404 | 347.972 | 291.991 | 403.954 | 454.098 | 431.398 | 476.798 |
| CXCL5 | 0.177 | 0.474 | -0.288 | 5532.783 | 4316.103 | 6749.463 | 3670.026 | 1695.601 | 5644.452 |
| CXCL9 | 0.188 | 0.474 | -0.262 | 232.427 | 170.038 | 294.815 | 141.591 | 88.938 | 194.244 |
| GDNF | 0.154 | 0.474 | 0.137 | 3.092 | 2.842 | 3.343 | 3.484 | 2.795 | 4.172 |
| sIL-10RB | 0.145 | 0.474 | 0.266 | 190.101 | 167.792 | 212.411 | 224.349 | 185.83 | 262.867 |
| sirtuin 2 | 0.177 | 0.474 | -0.331 | 29.413 | 20.683 | 38.144 | 17.796 | 11.124 | 24.469 |
| sOPG | 0.188 | 0.474 | 0.231 | 812.213 | 732.008 | 892.419 | 958.401 | 623.449 | 1293.353 |
| STAMBP | 0.16 | 0.474 | -0.221 | 14.011 | 11.859 | 16.164 | 11.205 | 8.729 | 13.681 |
| sTRANCE | 0.164 | 0.474 | 0.117 | 28.446 | 21.992 | 34.9 | 40.214 | 18.742 | 61.687 |
| VEGFA | 0.144 | 0.474 | -0.207 | 1407.973 | 1198.328 | 1617.617 | 1115.202 | 931.316 | 1299.088 |
| CXCL10 | 0.2 | 0.489 | -0.253 | 576.577 | 446.129 | 707.026 | 411.554 | 220.239 | 602.869 |
| CCL20 | 0.219 | 0.504 | -0.345 | 36.276 | 29.485 | 43.066 | 28.402 | 17.878 | 38.926 |
| sTWEAK | 0.227 | 0.509 | 0.301 | 329.624 | 288.094 | 371.154 | 385.839 | 276.134 | 495.543 |
| sSCF | 0.241 | 0.526 | 0.414 | 524.008 | 443.115 | 604.901 | 616.458 | 466.304 | 766.612 |
| CXCL1 | 0.308 | 0.639 | 0.035 | 278.396 | 242.94 | 313.853 | 317.601 | 209.243 | 425.96 |
| CCL2 | 0.342 | 0.66 | 0.338 | 987.117 | 888.382 | 1085.853 | 1504.389 | 487.813 | 2520.966 |
| CXCL11 | 0.342 | 0.66 | -0.288 | 140.781 | 112.36 | 169.201 | 105.71 | 91.181 | 120.239 |
| sTGFα | 0.332 | 0.66 | 0.082 | 9.991 | 8.37 | 11.612 | 11.708 | 7.67 | 15.745 |
| CCL3 | 0.363 | 0.685 | -0.312 | 9.891 | 7.853 | 11.928 | 6.565 | 5.646 | 7.485 |
| FGF19 | 0.419 | 0.772 | 0.4 | 168.278 | 122.995 | 213.56 | 235.598 | -20.936 | 492.132 |
| 4E-BP1 | 0.446 | 0.788 | 0.553 | 209.925 | 161.596 | 258.255 | 278.051 | 66.747 | 489.355 |
| NT3 | 0.441 | 0.788 | -0.12 | 2.581 | 2.354 | 2.807 | 2.453 | 1.409 | 3.498 |
| CCL13 | 0.493 | 0.809 | 0.014 | 10.429 | 8.909 | 11.949 | 11.863 | 6.912 | 16.815 |
| FGF5 | 0.543 | 0.809 | 0.044 | 2.324 | 2.106 | 2.542 | 2.793 | 1.245 | 4.342 |
| IL-10 | 0.523 | 0.809 | 0.275 | 12.925 | 11.471 | 14.379 | 16.006 | 4.827 | 27.184 |
| IL-17A | 0.521 | 0.809 | 0.046 | 1.078 | 1.007 | 1.15 | 1.242 | 0.567 | 1.916 |
| IL-18 | 0.531 | 0.809 | 0.424 | 126.893 | 102.665 | 151.12 | 150.19 | 60.949 | 239.432 |
| IL-7 | 0.546 | 0.809 | 0.057 | 27.991 | 24.07 | 31.912 | 24.303 | 13.38 | 35.227 |
| sCD244 | 0.49 | 0.809 | -0.071 | 74.331 | 67.839 | 80.823 | 67.574 | 43.001 | 92.148 |
| sSLAMF1 | 0.481 | 0.809 | 0.181 | 7.866 | 6.831 | 8.901 | 8.766 | 7.084 | 10.448 |
| sTNFβ | 0.543 | 0.809 | -0.141 | 11.078 | 9.268 | 12.887 | 10.281 | 5.681 | 14.882 |
| sCDCP1 | 0.56 | 0.816 | 0.249 | 6.882 | 5.813 | 7.952 | 7.811 | 2.975 | 12.647 |
| CCL19 | 0.574 | 0.822 | 0.058 | 519.736 | 396.086 | 643.386 | 421.735 | 170.685 | 672.785 |
| sIL-18R1 | 0.594 | 0.835 | 0.117 | 199.64 | 175.494 | 223.785 | 214.124 | 146.423 | 281.825 |
| IL-17C | 0.654 | 0.848 | -0.099 | 2.348 | 1.872 | 2.823 | 1.831 | 1.481 | 2.181 |
| IL-8 | 0.619 | 0.848 | 0.157 | 247.279 | 196.401 | 298.158 | 220.407 | 105.82 | 334.994 |
| sCD40 | 0.644 | 0.848 | 0.007 | 877.916 | 776.753 | 979.078 | 842.846 | 613.189 | 1072.502 |
| sCD5 | 0.652 | 0.848 | -0.078 | 11.918 | 10.599 | 13.236 | 11.149 | 7.016 | 15.282 |
| sCD6 | 0.644 | 0.848 | -0.129 | 10.891 | 9.461 | 12.322 | 10.701 | 3.738 | 17.664 |
| OSM | 0.682 | 0.871 | 0.054 | 29.209 | 23.283 | 35.136 | 27.62 | 23.211 | 32.028 |
| CCL8 | 0.722 | 0.908 | -0.117 | 1064.525 | 906.862 | 1222.189 | 976.174 | 539.971 | 1412.378 |
| CCL4 | 0.738 | 0.909 | 0.05 | 96.052 | 79.147 | 112.957 | 97.3 | 86.004 | 108.596 |
| CSF1 | 0.774 | 0.909 | 0.007 | 267.932 | 249.117 | 286.748 | 276.296 | 268.027 | 284.564 |
| CST5 | 0.766 | 0.909 | -0.084 | 84.904 | 78.896 | 90.913 | 83.875 | 55.719 | 112.03 |
| FGF23 | 0.784 | 0.909 | 0.007 | 3.459 | 2.941 | 3.977 | 3.875 | 1.978 | 5.773 |
| MMP10 | 0.788 | 0.909 | 0.04 | 489.223 | 423.249 | 555.196 | 489.511 | 411.918 | 567.103 |
| sIL-15RA | 0.749 | 0.909 | -0.067 | 1.704 | 1.564 | 1.844 | 1.822 | 1.237 | 2.407 |
| CCL23 | 0.837 | 0.952 | 0.052 | 649.857 | 558.341 | 741.374 | 615.804 | 296.398 | 935.21 |
| caspase 8 | 0.886 | 0.97 | 0 | 2.652 | 2.326 | 2.979 | 2.48 | 2.367 | 2.592 |
| CCL28 | 0.941 | 0.97 | -0.034 | 1.971 | 1.786 | 2.156 | 1.996 | 1.407 | 2.585 |
| CCL7 | 0.897 | 0.97 | 0.002 | 6.696 | 5.09 | 8.303 | 5.954 | 3.252 | 8.656 |
| IL-12B | 0.969 | 0.97 | -0.129 | 23.985 | 20.523 | 27.447 | 24.256 | 14.112 | 34.401 |
| IL-6 | 0.967 | 0.97 | 0.139 | 21.523 | 11.33 | 31.715 | 44.001 | -59.479 | 147.481 |
| LAP-TGFB1 | 0.871 | 0.97 | -0.027 | 175.962 | 156.57 | 195.354 | 171.719 | 141.87 | 201.568 |
| sADA | 0.97 | 0.97 | 0.117 | 24.593 | 22.237 | 26.95 | 24.316 | 19.751 | 28.881 |
| sHGF | 0.914 | 0.97 | -0.031 | 273.772 | 239.622 | 307.922 | 261.753 | 208.373 | 315.134 |
| uPA | 0.935 | 0.97 | 0.004 | 841.415 | 726.818 | 956.011 | 843.585 | 743.517 | 943.652 |

Legend: CI: 95% confidence interval

**Table S4: Regression analysis between cumulative glucocorticoid (GC) dose and other continuous clinical parameters.**

DAS28: 28-joint Disease Activity Score using C-reactive protein; GC: glucocorticoid; HAQ: health assessment questionnaire; SDAI: Simple Disease Activity Index.

| Dependent variable | Cumulative GC dose |  |  |  |  |  |  |
| --- | --- | --- | --- | --- | --- | --- | --- |
| Regression type | Least squares |  |  |  |  |  |  |
|  |  |  |  |  |  |  |  |
| Model |  |  |  |  |  |  |  |
| Analysis of Variance | Sum of squares | Degrees of freedom | Mean squared | F statistic (DFn, DFd) | P value |  |  |
| Regression | 1636 | 5 | 327.2 | F (5, 18) = 3.680 | P=0.0181 |  |  |
| Age | 319.4 | 1 | 319.4 | F (1, 18) = 3.592 | P=0.0742 |  |  |
| Disease duration | 452.1 | 1 | 452.1 | F (1, 18) = 5.084 | P=0.0368 |  |  |
| SDAI | 0.8325 | 1 | 0.8325 | F (1, 18) = 0.009363 | P=0.9240 |  |  |
| DAS28 | 5.456 | 1 | 5.456 | F (1, 18) = 0.06136 | P=0.8072 |  |  |
| HAQ | 176.3 | 1 | 176.3 | F (1, 18) = 1.982 | P=0.1762 |  |  |
| Residual | 1601 | 18 | 88.92 |  |  |  |  |
| Total | 3237 | 23 |  |  |  |  |  |
|  |  |  |  |  |  |  |  |
| Parameter estimates | Variable | Estimate | Standard error | 95% CI (asymptotic) | \|t\| | P value | P value summary |
| β0 | Intercept | -21.85 | 13.21 | -49.60 to 5.891 | 1.655 | 0.1153 | ns |
| β1 | Age | 0.3432 | 0.1811 | -0.03726 to 0.7236 | 1.895 | 0.0742 | ns |
| β2 | Disease duration | 0.8719 | 0.3867 | 0.05953 to 1.684 | 2.255 | 0.0368 | * |
| β3 | SDAI | -0.08239 | 0.8515 | -1.871 to 1.707 | 0.09676 | 0.924 | ns |
| β4 | DAS28 | 0.7774 | 3.138 | -5.816 to 7.371 | 0.2477 | 0.8072 | ns |
| β5 | HAQ | 4.354 | 3.093 | -2.143 to 10.85 | 1.408 | 0.1762 | ns |
|  |  |  |  |  |  |  |  |
| Goodness of Fit |  |  |  |  |  |  |  |
| Degrees of Freedom | 18 |  |  |  |  |  |  |
| R squared | 0.5055 |  |  |  |  |  |  |

**Table S5: Serum protein levels in active RA patients with low and high cumulative GC dose using a lower cut-off value of 15 g.**

|  |  |  |  | Low GC dose | | | High GC dose | | |
| --- | --- | --- | --- | --- | --- | --- | --- | --- | --- |
| Analyte | p value | Benjamini-Hochberg Correction | Fold Change | Mean | CI low | CI high | Mean | CI low | CI high |
| CCL20 | 0.000357 | 0.026386 | 0.694 | 106.413 | -38.326 | 251.151 | 90.066 | 51.49 | 128.642 |
| IL-17C | 0.002586 | 0.084618 | 0.316 | 1.924 | 1.619 | 2.23 | 3.418 | 2.243 | 4.594 |
| sOPG | 0.003498 | 0.084618 | 0.357 | 906.434 | 777.596 | 1035.271 | 1236.394 | 1054.111 | 1418.678 |
| IL-17A | 0.006248 | 0.084618 | 0.347 | 1.062 | 0.984 | 1.139 | 1.327 | 1.172 | 1.482 |
| sCD40 | 0.006651 | 0.084618 | 0.347 | 909.4 | 783.271 | 1035.529 | 1090.131 | 984.462 | 1195.801 |
| IL-18 | 0.006861 | 0.084618 | 0.395 | 147.429 | 104.921 | 189.937 | 195.908 | 162.181 | 229.636 |
| sIL-10RB | 0.008768 | 0.091867 | 0.319 | 181.659 | 165.927 | 197.392 | 221.616 | 198.915 | 244.317 |
| CSF1 | 0.010188 | 0.091867 | 0.11 | 274.313 | 253.816 | 294.81 | 315.572 | 295.076 | 336.068 |
| IL-8 | 0.012889 | 0.091867 | 0.505 | 270.411 | 198.838 | 341.984 | 671.392 | 37.402 | 1305.382 |
| MMP10 | 0.012889 | 0.091867 | 0.283 | 516.878 | 416.329 | 617.427 | 758.993 | 580.022 | 937.963 |
| sHGF | 0.013656 | 0.091867 | 0.292 | 320.992 | 279.261 | 362.724 | 440.051 | 367.792 | 512.311 |
| FGF21 | 0.021387 | 0.131884 | 1.329 | 25.146 | 14.71 | 35.582 | 67.296 | 32.578 | 102.014 |
| CCL25 | 0.027956 | 0.159135 | 0.248 | 81.151 | 66.73 | 95.572 | 124.975 | 97.684 | 152.267 |
| FGF5 | 0.033531 | 0.17409 | 0.166 | 2.354 | 2.139 | 2.57 | 2.8 | 2.452 | 3.148 |
| 4E-BP1 | 0.035288 | 0.17409 | 0.347 | 202.812 | 132.197 | 273.427 | 283.469 | 191.486 | 375.453 |
| sCDCP1 | 0.041041 | 0.18314 | 0.404 | 10.693 | 7.454 | 13.932 | 15.174 | 10.371 | 19.978 |
| FGF23 | 0.042073 | 0.18314 | 0.159 | 4.298 | 3.354 | 5.242 | 9.944 | 2.898 | 16.991 |
| CXCL9 | 0.060379 | 0.235158 | 0.154 | 1.753 | 1.575 | 1.93 | 1.937 | 1.745 | 2.129 |
| sIL-15RA | 0.060379 | 0.235158 | 0.31 | 262.607 | 209.34 | 315.873 | 491.049 | 261.473 | 720.625 |
| CCL3 | 0.079397 | 0.292465 | 0.265 | 7.59 | 6.081 | 9.098 | 10.367 | 7.657 | 13.077 |
| sCX3CL1 | 0.082997 | 0.292465 | 0.079 | 86.845 | 69.013 | 104.678 | 94.666 | 84.773 | 104.558 |
| CCL28 | 0.094595 | 0.308655 | 0.142 | 2.13 | 1.889 | 2.372 | 2.462 | 2.181 | 2.743 |
| bNGF | 0.107457 | 0.308655 | 0.103 | 2.809 | 2.584 | 3.034 | 3.33 | 2.802 | 3.858 |
| sTRANCE | 0.107457 | 0.308655 | -0.258 | 35.188 | 28.445 | 41.931 | 27.532 | 21.403 | 33.662 |
| caspase 8 | 0.116775 | 0.308655 | 0.173 | 2.717 | 2.363 | 3.07 | 3.486 | 2.645 | 4.328 |
| OSM | 0.124171 | 0.308655 | 0.329 | 38.79 | 29.938 | 47.642 | 47.704 | 39.42 | 55.989 |
| uPA | 0.126716 | 0.308655 | 0.197 | 13.84 | 10.752 | 16.927 | 18.217 | 14.22 | 22.214 |
| sTGFα | 0.126716 | 0.308655 | 0.149 | 841.311 | 735.579 | 947.043 | 939.028 | 839.386 | 1038.67 |
| CCL11 | 0.126716 | 0.308655 | 0.157 | 360.211 | 326.568 | 393.854 | 419.696 | 360.336 | 479.057 |
| CXCL1 | 0.129302 | 0.308655 | 0.072 | 9.519 | 8.578 | 10.46 | 10.334 | 9.496 | 11.172 |
| sLIFR | 0.129302 | 0.308655 | 0.11 | 353.929 | 309.59 | 398.268 | 421.7 | 366.584 | 476.815 |
| sTRAIL | 0.134595 | 0.311252 | -0.092 | 269.107 | 242.086 | 296.127 | 247.799 | 222.526 | 273.072 |
| sFlt3L | 0.154449 | 0.342702 | 0.214 | 516.445 | 420.735 | 612.155 | 596.84 | 506.523 | 687.157 |
| sPDL1 | 0.157458 | 0.342702 | 0.133 | 31.22 | 25.579 | 36.861 | 32.114 | 28.911 | 35.318 |
| STAMBP | 0.173173 | 0.366136 | 0.197 | 12.182 | 10.285 | 14.079 | 13.969 | 11.761 | 16.176 |
| sTNFSF14 | 0.179777 | 0.367237 | 0.133 | 56.033 | 41.284 | 70.783 | 63.461 | 50.921 | 76.001 |
| VEGFA | 0.186566 | 0.367237 | 0.094 | 1785.848 | 1448.632 | 2123.064 | 2064.58 | 1750.44 | 2378.721 |
| GDNF | 0.190031 | 0.367237 | 0.086 | 3.718 | 3.261 | 4.174 | 4.794 | 3.331 | 6.256 |
| sCD244 | 0.193544 | 0.367237 | 0.125 | 66.182 | 57.441 | 74.922 | 72.964 | 64.308 | 81.621 |
| CCL4 | 0.200711 | 0.371315 | 0.094 | 81.002 | 70.211 | 91.793 | 104.975 | 81.044 | 128.905 |
| sSLAMF1 | 0.215622 | 0.389171 | 0.173 | 8.504 | 6.79 | 10.218 | 12.116 | 6.646 | 17.586 |
| IL-6 | 0.22337 | 0.393556 | 1.313 | 117.898 | 4.438 | 231.358 | 116.015 | 47.563 | 184.467 |
| CCL23 | 0.243605 | 0.419227 | 0.094 | 659.623 | 575.172 | 744.075 | 746.252 | 651.106 | 841.398 |
| sTWEAK | 0.260696 | 0.433616 | 0.102 | 332.863 | 301.03 | 364.697 | 359.532 | 321.621 | 397.443 |
| axin-1 | 0.265095 | 0.433616 | 0.206 | 2.757 | 2.147 | 3.366 | 3.264 | 2.506 | 4.021 |
| CCL13 | 0.269545 | 0.433616 | -0.027 | 12.664 | 10.091 | 15.237 | 10.96 | 8.984 | 12.935 |
| IL-10 | 0.29732 | 0.468121 | 0.125 | 18.058 | 13.003 | 23.114 | 18.958 | 14.968 | 22.948 |
| sSCF | 0.326946 | 0.504041 | -0.105 | 522.723 | 456.928 | 588.519 | 473.939 | 410.258 | 537.619 |
| SULT1A1 | 0.337233 | 0.50929 | 0.79 | 14.765 | 9.372 | 20.158 | 17.222 | 12.101 | 22.343 |
| sirtuin 2 | 0.358424 | 0.530468 | 0.515 | 23.164 | 15.855 | 30.473 | 26.582 | 19.076 | 34.088 |
| BDNF | 0.409086 | 0.593576 | -0.371 | 113.603 | 23.288 | 203.918 | 72.974 | -34.205 | 180.152 |
| CCL2 | 0.445111 | 0.633428 | 0.035 | 1281.137 | 1066.88 | 1495.394 | 1489.04 | 1131.11 | 1846.971 |
| sADA | 0.463782 | 0.647544 | 0.08 | 27.615 | 22.506 | 32.725 | 27.173 | 24.76 | 29.587 |
| CXCL10 | 0.529057 | 0.725004 | 0.035 | 768.646 | 585.67 | 951.622 | 995.089 | 658.043 | 1332.134 |
| sIL-18R1 | 0.549513 | 0.739345 | 0.035 | 235.413 | 196.156 | 274.671 | 241.159 | 209.484 | 272.834 |
| CXCL11 | 0.598709 | 0.770683 | 0.035 | 168.596 | 141.575 | 195.616 | 219.684 | 139.231 | 300.137 |
| CXCL6 | 0.620387 | 0.770683 | -0.147 | 190.329 | 141.598 | 239.06 | 209.914 | 157.617 | 262.211 |
| FGF19 | 0.620387 | 0.770683 | 0.007 | 457.608 | 383.749 | 531.467 | 472.096 | 329.085 | 615.106 |
| LAP-TGFB1 | 0.6424 | 0.770683 | 0.301 | 180.557 | 157.099 | 204.014 | 189.102 | 166.583 | 211.622 |
| MMP1 | 0.6424 | 0.770683 | 0.165 | 1509.095 | 1011.81 | 2006.38 | 1426.848 | 1144.051 | 1709.645 |
| IL-7 | 0.657254 | 0.770683 | -0.099 | 31.459 | 27.217 | 35.7 | 30.662 | 26.108 | 35.216 |
| IL-12B | 0.664732 | 0.770683 | -0.086 | 11.657 | 8.689 | 14.626 | 14.674 | 6.902 | 22.446 |
| sTNFβ | 0.664732 | 0.770683 | -0.182 | 24.344 | 18.401 | 30.287 | 22.736 | 17.233 | 28.238 |
| CXCL5 | 0.679789 | 0.770683 | -0.027 | 158.293 | 146.821 | 169.764 | 154.565 | 143.76 | 165.37 |
| sDNER | 0.679789 | 0.770683 | -0.041 | 5689.61 | 4737.91 | 6641.31 | 5461.908 | 4589.859 | 6333.956 |
| CCL19 | 0.687366 | 0.770683 | -0.165 | 770.287 | 551.599 | 988.976 | 865.723 | 479.174 | 1252.272 |
| NT3 | 0.710283 | 0.784491 | 0.008 | 2.659 | 2.375 | 2.943 | 3.74 | 1.774 | 5.706 |
| sTNFRSF9 | 0.804405 | 0.875381 | 0.079 | 108.381 | 89.436 | 127.326 | 108.884 | 90.261 | 127.507 |
| CST5 | 0.85265 | 0.914436 | 0.064 | 113.361 | 92.392 | 134.331 | 108.441 | 92.116 | 124.766 |
| CCL7 | 0.909629 | 0.961608 | -0.054 | 15.319 | 10.013 | 20.624 | 16.352 | 9.473 | 23.231 |
| sCD5 | 0.958852 | 0.988665 | -0.007 | 12.498 | 11.068 | 13.928 | 12.709 | 11.06 | 14.359 |
| CCL8 | 0.967077 | 0.988665 | 0.014 | 1182.43 | 983.145 | 1381.715 | 1146.418 | 988.615 | 1304.22 |
| sCD6 | 0.975304 | 0.988665 | -0.027 | 10.249 | 8.861 | 11.637 | 11.172 | 9.262 | 13.082 |
| EN-RAGE | 0.991767 | 0.991767 | -0.034 | 80.225 | 60.089 | 100.361 | 86.106 | 59.937 | 112.276 |

Legend: CI: 95% confidence interval

**Table S6: Serum levels of studied proteins in patients with low and high GC dose within active RA group when using DAS28.**

DAS28: 28-joint Disease Activity Score using C-reactive protein.

|  |  |  |  | Low GC dose | | | High GC dose | | |
| --- | --- | --- | --- | --- | --- | --- | --- | --- | --- |
| Analyte | p value | Benjamini-Hochberg Correction | Fold Change | Mean | CI low | CI high | Mean | CI low | CI high |
| sHGF | 0 | 0.005 | 0.79 | 283.776 | 247.591 | 319.96 | 500.944 | 409.746 | 592.141 |
| OSM | 0 | 0.005 | 0.959 | 28.885 | 22.869 | 34.901 | 54.994 | 45.053 | 64.934 |
| sTGFα | 0 | 0.005 | 0.803 | 9.761 | 8.028 | 11.493 | 21.221 | 16.004 | 26.439 |
| 4E-BP1 | 0 | 0.005 | 0.796 | 143.303 | 115.44 | 171.167 | 298.226 | 218.361 | 378.091 |
| caspase 8 | 0.001 | 0.02 | 0.324 | 2.414 | 2.142 | 2.686 | 3.538 | 2.959 | 4.117 |
| sLIFR | 0.002 | 0.02 | 0.165 | 8.459 | 7.944 | 8.975 | 10.017 | 9.22 | 10.813 |
| sCD40 | 0.002 | 0.02 | 0.301 | 878.497 | 791.153 | 965.841 | 1163.598 | 1024.07 | 1303.126 |
| STAMBP | 0.003 | 0.026 | 0.31 | 10.96 | 9.437 | 12.482 | 15.789 | 13.093 | 18.486 |
| uPA | 0.003 | 0.026 | 0.261 | 798.42 | 712.614 | 884.226 | 998.848 | 910.172 | 1087.524 |
| sIL-10RB | 0.004 | 0.026 | 0.347 | 180.089 | 165.602 | 194.576 | 228.097 | 201.338 | 254.856 |
| LAP-TGFB1 | 0.004 | 0.028 | 0.434 | 154.022 | 135.607 | 172.437 | 206.658 | 179.079 | 234.237 |
| IL-18 | 0.006 | 0.036 | 0.486 | 156.152 | 116.811 | 195.492 | 212.004 | 179.237 | 244.771 |
| IL-8 | 0.007 | 0.038 | 0.388 | 241.785 | 206.409 | 277.16 | 328.316 | 279.322 | 377.311 |
| sTNFSF14 | 0.007 | 0.04 | 0.39 | 45.217 | 37.174 | 53.259 | 63.821 | 53.923 | 73.718 |
| sOPG | 0.015 | 0.072 | 0.474 | 963.096 | 804.297 | 1121.895 | 1297.339 | 1063.685 | 1530.994 |
| sIL-18R1 | 0.015 | 0.072 | 0.231 | 207.786 | 181.777 | 233.796 | 244.556 | 232.343 | 256.769 |
| sirtuin 2 | 0.017 | 0.074 | 0.636 | 18.148 | 14.053 | 22.242 | 28.909 | 20.79 | 37.029 |
| CCL25 | 0.019 | 0.078 | 1.014 | 77.328 | 64.434 | 90.222 | 137.083 | 99.502 | 174.664 |
| CCL20 | 0.022 | 0.081 | 0.521 | 44.998 | 36.706 | 53.29 | 65.96 | 49.922 | 81.998 |
| FGF5 | 0.022 | 0.081 | 0.209 | 2.333 | 2.097 | 2.57 | 2.814 | 2.402 | 3.227 |
| sCDCP1 | 0.023 | 0.081 | 0.527 | 8.714 | 7.037 | 10.39 | 15.488 | 10.225 | 20.75 |
| VEGFA | 0.024 | 0.082 | 0.231 | 1586.978 | 1357.535 | 1816.421 | 2244.69 | 1813.456 | 2675.924 |
| axin-1 | 0.026 | 0.083 | 0.375 | 2.352 | 1.961 | 2.742 | 3.304 | 2.559 | 4.048 |
| sCX3CL1 | 0.028 | 0.088 | 0.151 | 75.069 | 67.838 | 82.3 | 91.259 | 81.519 | 100.999 |
| IL-17A | 0.033 | 0.094 | 0.277 | 1.095 | 1.012 | 1.178 | 1.312 | 1.156 | 1.469 |
| MMP1 | 0.034 | 0.094 | 0.705 | 1098.341 | 777.728 | 1418.954 | 1591.792 | 1268.301 | 1915.283 |
| CXCL9 | 0.036 | 0.094 | 0.424 | 265.003 | 214.77 | 315.235 | 404.945 | 281.103 | 528.788 |
| CCL8 | 0.036 | 0.094 | 0.283 | 975.331 | 801.039 | 1149.623 | 1168.953 | 1016.888 | 1321.017 |
| SULT1A1 | 0.037 | 0.094 | 1.057 | 11.617 | 7.656 | 15.577 | 20.199 | 13.33 | 27.068 |
| sPDL1 | 0.046 | 0.11 | 0.216 | 27.739 | 24.245 | 31.234 | 33.384 | 29.475 | 37.292 |
| CCL11 | 0.048 | 0.11 | 0.189 | 349.294 | 305.309 | 393.279 | 433.511 | 360.626 | 506.396 |
| sFlt3L | 0.048 | 0.11 | 0.292 | 484.787 | 411.004 | 558.571 | 629.754 | 512.42 | 747.087 |
| EN-RAGE | 0.051 | 0.115 | 0.486 | 56.333 | 41.724 | 70.943 | 89.975 | 61.998 | 117.952 |
| sTWEAK | 0.055 | 0.119 | 0.197 | 318.076 | 285.598 | 350.554 | 368.59 | 316.084 | 421.096 |
| IL-10 | 0.058 | 0.122 | 0.394 | 14.969 | 11.699 | 18.238 | 19.524 | 15.109 | 23.94 |
| CXCL1 | 0.062 | 0.128 | 0.137 | 351.306 | 303.188 | 399.424 | 430.526 | 368.389 | 492.663 |
| CCL3 | 0.066 | 0.132 | 0.481 | 7.708 | 6.186 | 9.231 | 9.765 | 7.939 | 11.591 |
| CCL23 | 0.073 | 0.143 | 0.385 | 645.964 | 568.024 | 723.903 | 797.169 | 673.531 | 920.808 |
| IL-17C | 0.076 | 0.145 | 0.181 | 2.027 | 1.734 | 2.32 | 2.73 | 2.045 | 3.415 |
| bNGF | 0.091 | 0.169 | 0.09 | 2.763 | 2.479 | 3.047 | 3.134 | 2.782 | 3.487 |
| FGF21 | 0.096 | 0.17 | 1.329 | 26.454 | 14.464 | 38.444 | 44.483 | 25.346 | 63.619 |
| CSF1 | 0.097 | 0.17 | 0.042 | 289.058 | 263.248 | 314.869 | 318.951 | 295.751 | 342.151 |
| IL-6 | 0.109 | 0.188 | 1.047 | 34.738 | 22.432 | 47.043 | 120.828 | 49.835 | 191.821 |
| CCL19 | 0.116 | 0.194 | -0.298 | 781.358 | 561.25 | 1001.466 | 496.772 | 398.657 | 594.886 |
| GDNF | 0.139 | 0.229 | 0.102 | 3.431 | 3.163 | 3.7 | 4.213 | 3.479 | 4.947 |
| CXCL10 | 0.147 | 0.237 | 0.307 | 754.722 | 593.278 | 916.166 | 1041.637 | 734.153 | 1349.12 |
| CCL28 | 0.156 | 0.246 | 0.082 | 2.14 | 1.881 | 2.399 | 2.499 | 2.163 | 2.835 |
| FGF23 | 0.16 | 0.246 | 0.169 | 3.943 | 3.289 | 4.597 | 4.517 | 3.604 | 5.43 |
| MMP10 | 0.179 | 0.27 | 0.102 | 573.552 | 437.205 | 709.899 | 616.508 | 518.849 | 714.167 |
| CXCL11 | 0.203 | 0.3 | 0.057 | 165.218 | 135.395 | 195.04 | 212.142 | 163.995 | 260.289 |
| FGF19 | 0.214 | 0.305 | 0.027 | 149.586 | 114.227 | 184.946 | 193.099 | 144.2 | 241.999 |
| CCL2 | 0.214 | 0.305 | 0.125 | 1195.036 | 1016.374 | 1373.697 | 1427.818 | 1151.087 | 1704.549 |
| CCL7 | 0.227 | 0.317 | 0.084 | 11.557 | 8.198 | 14.916 | 16.69 | 10.079 | 23.301 |
| sIL-15RA | 0.231 | 0.317 | 0.109 | 1.727 | 1.585 | 1.87 | 1.855 | 1.664 | 2.045 |
| sSCF | 0.278 | 0.374 | 0.157 | 464.791 | 397.781 | 531.801 | 511.087 | 448.735 | 573.439 |
| sSLAMF1 | 0.297 | 0.392 | 0.138 | 8.375 | 6.963 | 9.787 | 9.914 | 7.504 | 12.323 |
| sTNFβ | 0.317 | 0.411 | -0.136 | 10.188 | 8.904 | 11.472 | 9.164 | 7.497 | 10.832 |
| sCD6 | 0.325 | 0.415 | -0.089 | 10.227 | 8.902 | 11.553 | 9.501 | 7.979 | 11.023 |
| CCL4 | 0.364 | 0.457 | 0.138 | 85.972 | 73.941 | 98.004 | 104.657 | 78.546 | 130.769 |
| CXCL6 | 0.381 | 0.47 | 0.102 | 389.68 | 328.945 | 450.416 | 422.808 | 355.396 | 490.219 |
| sCD244 | 0.412 | 0.5 | 0.087 | 66.039 | 57.735 | 74.343 | 68.185 | 62.782 | 73.588 |
| CST5 | 0.426 | 0.508 | -0.083 | 124.236 | 100.245 | 148.226 | 102.271 | 88.956 | 115.587 |
| sCD5 | 0.497 | 0.584 | -0.07 | 12.495 | 11.097 | 13.893 | 11.943 | 9.988 | 13.898 |
| sADA | 0.63 | 0.728 | 0.043 | 25.856 | 23.34 | 28.372 | 27.339 | 24.335 | 30.344 |
| IL-12B | 0.641 | 0.73 | 0.308 | 21.021 | 15.094 | 26.947 | 21.046 | 16.285 | 25.808 |
| CXCL5 | 0.703 | 0.789 | 0.068 | 4945.141 | 4249.021 | 5641.26 | 5213.884 | 4308.443 | 6119.325 |
| CCL13 | 0.745 | 0.82 | 0.017 | 11.012 | 9.371 | 12.654 | 9.961 | 8.305 | 11.618 |
| sTNFRSF9 | 0.753 | 0.82 | 0.035 | 103.219 | 85.208 | 121.231 | 107.966 | 88.042 | 127.891 |
| sTRANCE | 0.8 | 0.853 | -0.124 | 29.048 | 22.791 | 35.306 | 30.575 | 22.206 | 38.943 |
| IL-7 | 0.807 | 0.853 | 0.014 | 31.392 | 26.624 | 36.159 | 32.371 | 26.36 | 38.382 |
| sTRAIL | 0.854 | 0.891 | -0.051 | 250.55 | 219.772 | 281.328 | 258.649 | 235.983 | 281.315 |
| BDNF | 0.95 | 0.976 | 0.178 | 10.534 | 2.027 | 19.041 | 6.623 | 1.712 | 11.533 |
| sDNER | 0.973 | 0.987 | -0.024 | 151.892 | 138.337 | 165.447 | 153.129 | 142.701 | 163.556 |
| NT3 | 1 | 1 | 0.016 | 2.59 | 2.311 | 2.869 | 2.528 | 2.22 | 2.836 |

Legend: CI: 95% confidence interval

**Table S7: Serum levels of studied proteins in patients with RA when compared to control group of healthy individuals.**

|  |  |  |  |  | RA |  |  | Control group |  |
| --- | --- | --- | --- | --- | --- | --- | --- | --- | --- |
| Analyte | p value | Benjamini-Hochberg Correction | Fold Change | Mean | CI low | CI high | Mean | CI low | CI high |
| SULT1A1 | 8.95E-13 | 3.89275E-11 | -0.87973417 | 17.03139914 | 13.91445241 | 20.14834587 | 1.685783578 | 1.443658509 | 1.927908647 |
| sTNFSF14 | 1.05E-12 | 3.89275E-11 | -0.781548309 | 53.31371193 | 48.85289494 | 57.77452891 | 12.41792957 | 10.79059741 | 14.04526173 |
| axin-1 | 3.19E-10 | 7.87688E-09 | -0.484310174 | 2.75477887 | 2.477671174 | 3.031886566 | 1.255735726 | 1.183630644 | 1.327840808 |
| IL-6 | 1.25E-09 | 2.31096E-08 | -0.75853912 | 32.93566678 | 24.32600816 | 41.54532541 | 5.339318648 | 4.404833867 | 6.27380343 |
| OSM | 2.97E-09 | 4.06478E-08 | -0.557769135 | 37.69328081 | 33.5333258 | 41.85323582 | 15.46906138 | 12.17993696 | 18.75818579 |
| sHGF | 3.3E-09 | 4.06478E-08 | -0.35939401 | 320.6743649 | 297.1360658 | 344.212664 | 192.71592 | 179.2497315 | 206.1821084 |
| caspase 8 | 4.1E-09 | 4.33169E-08 | -0.38014615 | 2.708997708 | 2.529598477 | 2.888396938 | 1.715876595 | 1.575113525 | 1.856639665 |
| CCL7 | 2.23E-08 | 2.06014E-07 | -0.530238625 | 9.354625823 | 7.950028973 | 10.75922267 | 4.14074648 | 3.525597734 | 4.755895226 |
| CCL3 | 4.24E-08 | 3.48899E-07 | -0.396510696 | 8.586782058 | 7.706408141 | 9.467155974 | 4.500388702 | 3.970317287 | 5.030460118 |
| sTGFα | 7.37E-08 | 5.45196E-07 | -0.456645622 | 12.07482981 | 10.95359389 | 13.19606572 | 6.275070074 | 5.090427064 | 7.459713083 |
| sCD40 | 1.74E-07 | 1.17289E-06 | -0.307445266 | 947.4480757 | 888.482605 | 1006.413546 | 642.7516794 | 582.6354474 | 702.8679114 |
| STAMBP | 4.54E-07 | 2.80038E-06 | -0.353998095 | 12.87195366 | 11.8563493 | 13.88755803 | 8.365660068 | 7.693622474 | 9.037697662 |
| sirtuin 2 | 5.82E-07 | 3.31076E-06 | -0.465899405 | 21.11997811 | 18.50862723 | 23.73132899 | 10.49807468 | 9.105212782 | 11.89093658 |
| EN-RAGE | 1.46E-06 | 7.72262E-06 | -0.597777846 | 60.15553082 | 51.67428061 | 68.63678103 | 24.31480633 | 19.06995375 | 29.55965891 |
| FGF23 | 3.76E-05 | 0.000185616 | -0.250090311 | 3.82183542 | 3.574013643 | 4.069657197 | 2.849493302 | 2.62331409 | 3.075672514 |
| CXCL10 | 6.18E-05 | 0.000285717 | -0.435072712 | 673.2718642 | 584.9037329 | 761.6399954 | 382.4160268 | 295.9360749 | 468.8959787 |
| sSLAMF1 | 0.000107 | 0.000466685 | -0.22083542 | 8.271581244 | 7.58191855 | 8.961243939 | 6.099148778 | 5.639583993 | 6.558713563 |
| sTRANCE | 0.000781 | 0.00321233 | -0.409532039 | 32.14346897 | 28.40607453 | 35.8808634 | 20.23664944 | 16.862319 | 23.61097987 |
| CXCL11 | 0.001222 | 0.004761176 | -0.340182647 | 154.87447 | 140.1569733 | 169.5919668 | 107.8088028 | 85.78480771 | 129.8327979 |
| sIL-18R1 | 0.001729 | 0.006397953 | -0.2028932 | 213.2171013 | 201.498823 | 224.9353796 | 179.3047498 | 162.848524 | 195.7609756 |
| MMP1 | 0.002314 | 0.008154141 | -0.317188095 | 1250.352523 | 1089.417449 | 1411.287598 | 777.8695781 | 640.9673614 | 914.7717949 |
| CST5 | 0.002887 | 0.00970973 | 0.265756594 | 91.90963674 | 86.49395548 | 97.32531801 | 123.046091 | 103.3867378 | 142.7054443 |
| FGF19 | 0.003473 | 0.011173599 | 0.421628214 | 184.532596 | 160.1192682 | 208.9459239 | 257.7559478 | 212.6886874 | 302.8232081 |
| CXCL1 | 0.004615 | 0.014230469 | -0.247376626 | 347.9535161 | 323.9415572 | 371.965475 | 276.1184535 | 234.1960937 | 318.0408133 |
| CXCL9 | 0.005576 | 0.016503587 | -0.292825271 | 265.6080555 | 233.370828 | 297.8452829 | 182.2786209 | 148.5084112 | 216.0488305 |
| IL-7 | 0.007799 | 0.022197797 | 0.27438323 | 28.71400165 | 26.6575748 | 30.7704285 | 40.04268773 | 32.29906163 | 47.78631383 |
| IL-10 | 0.010199 | 0.027953343 | -0.237571994 | 14.42718347 | 13.23316099 | 15.62120595 | 11.74041492 | 10.15393755 | 13.32689228 |
| sTNFRSF9 | 0.011727 | 0.030993383 | -0.225324848 | 102.0873635 | 93.88705496 | 110.287672 | 82.8484573 | 72.96456436 | 92.73235025 |
| CCL4 | 0.015635 | 0.039210094 | -0.22083542 | 90.06548897 | 82.42285749 | 97.70812046 | 72.64026096 | 64.35669081 | 80.92383112 |
| IL-8 | 0.015896 | 0.039210094 | -0.141551194 | 259.1305792 | 233.7647302 | 284.4964282 | 202.0483732 | 177.385083 | 226.7116633 |
| sTRAIL | 0.019832 | 0.04734026 | -0.142339411 | 249.4999791 | 235.7062547 | 263.2937034 | 225.6385847 | 207.0514877 | 244.2256817 |
| CXCL6 | 0.02555 | 0.059085355 | -0.203660727 | 413.8838709 | 381.8882541 | 445.8794877 | 337.4854287 | 283.9083162 | 391.0625411 |
| sIL-15RA | 0.035451 | 0.079496539 | -0.089193434 | 1.767977298 | 1.688193386 | 1.847761211 | 1.594113281 | 1.45716581 | 1.731060752 |
| IL-18 | 0.039539 | 0.086056485 | -0.262759142 | 151.2205118 | 135.1204211 | 167.3206026 | 118.1723931 | 99.58378572 | 136.7610006 |
| LAP-TGFB1 | 0.041627 | 0.088010374 | -0.053942353 | 178.4312782 | 167.6055917 | 189.2569646 | 155.5037231 | 141.1434246 | 169.8640216 |
| CCL2 | 0.053439 | 0.109845913 | -0.204463516 | 1262.669745 | 1150.084575 | 1375.254915 | 1058.084476 | 915.7246645 | 1200.444288 |
| CCL13 | 0.057092 | 0.114184421 | -0.177538775 | 10.57120295 | 9.814608421 | 11.32779748 | 9.229165697 | 8.168860795 | 10.2894706 |
| sPDL1 | 0.066572 | 0.129641042 | -0.156189272 | 29.72883438 | 28.05900637 | 31.39866239 | 27.06081944 | 24.36381219 | 29.75782668 |
| sTNFβ | 0.07021 | 0.133218181 | 0.190607752 | 10.29249309 | 9.466330689 | 11.11865549 | 11.45227503 | 10.32247062 | 12.58207944 |
| sCDCP1 | 0.074171 | 0.137216407 | -0.137468885 | 8.570602649 | 7.705884972 | 9.435320326 | 7.270579135 | 6.135329216 | 8.405829054 |
| VEGFA | 0.077938 | 0.140668129 | -0.154930252 | 1649.982774 | 1500.036516 | 1799.929031 | 1369.701218 | 1191.62193 | 1547.780507 |
| BDNF | 0.125333 | 0.220825038 | 13.28181251 | 17.13805863 | 9.556617146 | 24.71950012 | 70.72900818 | 23.89139814 | 117.5666182 |
| sOPG | 0.137216 | 0.236138414 | 0.105724013 | 940.3337369 | 872.1572418 | 1008.510232 | 1009.425321 | 917.95014 | 1100.900502 |
| GDNF | 0.150057 | 0.252368467 | 0.060673633 | 3.396751585 | 3.241474097 | 3.552029073 | 3.681057519 | 3.330276183 | 4.031838854 |
| NT3 | 0.21081 | 0.346664573 | 0.05471588 | 2.595423092 | 2.457294326 | 2.733551858 | 2.853715575 | 2.533555322 | 3.173875829 |
| sDNER | 0.248137 | 0.39917671 | 0.028949745 | 155.0408944 | 148.935655 | 161.1461337 | 163.1582663 | 151.0070503 | 175.3094822 |
| sCD6 | 0.28861 | 0.454407263 | -0.104974929 | 10.35425704 | 9.559641804 | 11.14887228 | 9.466496271 | 8.443684088 | 10.48930845 |
| FGF21 | 0.29546 | 0.455500814 | -0.236870396 | 27.1335533 | 21.24588764 | 33.02121895 | 16.17987778 | 12.89793743 | 19.46181813 |
| sSCF | 0.306379 | 0.462695121 | 0.0460284 | 512.6332333 | 475.8238021 | 549.4426645 | 544.5221463 | 493.3194584 | 595.7248342 |
| FGF5 | 0.319925 | 0.4734886 | 0.062145109 | 2.415121759 | 2.29195772 | 2.538285798 | 2.543751664 | 2.352277582 | 2.735225747 |
| sFlt3L | 0.350926 | 0.509187033 | 0.117287138 | 487.3580584 | 447.4690689 | 527.2470478 | 499.7799951 | 463.0040973 | 536.5558928 |
| sCD5 | 0.36203 | 0.512034557 | -0.105168402 | 12.06146808 | 11.3480508 | 12.77488537 | 11.41693921 | 10.37667282 | 12.4572056 |
| CCL19 | 0.366915 | 0.512034557 | -0.073411938 | 592.1485487 | 517.8482272 | 666.4488701 | 518.8652586 | 421.8387232 | 615.8917941 |
| IL-12B | 0.373647 | 0.512034557 | -0.098706113 | 21.60482132 | 19.56844371 | 23.64119893 | 19.78072881 | 17.11851351 | 22.44294411 |
| CCL25 | 0.404357 | 0.543001681 | 0 | 86.35094332 | 77.2333375 | 95.46854913 | 94.66754611 | 78.46703161 | 110.8680606 |
| CCL23 | 0.41092 | 0.543001681 | -0.089324697 | 693.1620277 | 642.1681355 | 744.1559199 | 653.689025 | 558.577605 | 748.8004449 |
| CCL8 | 0.442401 | 0.566152599 | -0.084764984 | 1109.195539 | 1021.213838 | 1197.17724 | 1041.531614 | 943.2213623 | 1139.841866 |
| CSF1 | 0.443741 | 0.566152599 | 0.010457515 | 287.3082825 | 275.8217985 | 298.7947664 | 303.1640631 | 268.0135051 | 338.314621 |
| CCL28 | 0.463923 | 0.581868992 | -0.009021354 | 2.197691008 | 2.058538179 | 2.336843836 | 2.310229283 | 2.064775563 | 2.555683003 |
| CCL11 | 0.536687 | 0.661913648 | -0.092480845 | 378.8318591 | 352.3107402 | 405.352978 | 361.9702053 | 320.4065297 | 403.5338809 |
| bNGF | 0.556055 | 0.674558886 | -0.006909529 | 2.86049512 | 2.733047827 | 2.987942413 | 2.735106086 | 2.517614987 | 2.952597185 |
| sADA | 0.656297 | 0.78332203 | -0.00748442 | 25.53918847 | 24.2460611 | 26.83231583 | 26.1295534 | 24.08122469 | 28.1778821 |
| CXCL5 | 0.693831 | 0.813035284 | 0.013041631 | 5200.299325 | 4722.426233 | 5678.172417 | 5604.254456 | 4645.485555 | 6563.023357 |
| IL-17A | 0.703166 | 0.813035284 | -0.092354487 | 1.158235373 | 1.098341613 | 1.218129133 | 1.220102402 | 1.055645783 | 1.384559021 |
| CCL20 | 0.721714 | 0.821643614 | -0.124491248 | 43.64312789 | 38.87986603 | 48.40638975 | 46.96261439 | 33.99817353 | 59.92705526 |
| sCD244 | 0.74845 | 0.839171335 | 0.003465722 | 68.60661768 | 64.82351218 | 72.38972319 | 69.4480224 | 63.36899367 | 75.52705114 |
| 4E-BP1 | 0.772495 | 0.853203122 | 0.05449253 | 202.0664367 | 176.1474257 | 227.9854478 | 196.443457 | 167.2975755 | 225.5893384 |
| sLIFR | 0.818388 | 0.877883274 | 0.064196052 | 9.674544786 | 9.25129288 | 10.09779669 | 9.605518531 | 9.095711708 | 10.11532535 |
| uPA | 0.826057 | 0.877883274 | 0.038703147 | 857.521172 | 810.0302385 | 905.0121055 | 858.4666051 | 785.5130979 | 931.4201123 |
| sCX3CL1 | 0.83043 | 0.877883274 | -0.015886805 | 88.33209434 | 82.66967714 | 93.99451155 | 90.09919852 | 79.59312833 | 100.6052687 |
| MMP10 | 0.873889 | 0.910813932 | 0.046078657 | 534.7428906 | 489.5394109 | 579.9463703 | 530.8468517 | 444.4472191 | 617.2464844 |
| sIL-10RB | 0.960526 | 0.969695245 | 0.0460284 | 200.7252395 | 189.2834779 | 212.1670011 | 201.6705778 | 179.7042012 | 223.6369543 |
| sTWEAK | 0.967102 | 0.969695245 | 0.00695555 | 344.9473274 | 325.0698991 | 364.8247557 | 342.0368731 | 313.0646731 | 371.0090732 |
| IL-17C | 0.969695 | 0.969695245 | -0.012664249 | 2.157856924 | 1.970804867 | 2.344908982 | 2.040150898 | 1.823073735 | 2.257228061 |

Legend: CI: 95% confidence interval

**Figure S1: Distribution of serum levels of proteins different between RA patients with high cumulative GC dose (High GC) when compared to the low cumulative GC dose group (Low GC) within active RA.** This figure does not show the highest ranked proteins identified in PCA, which are shown in Figure 1.

Group means are indicated by horizontal bars, error bars indicate 95 % CI.


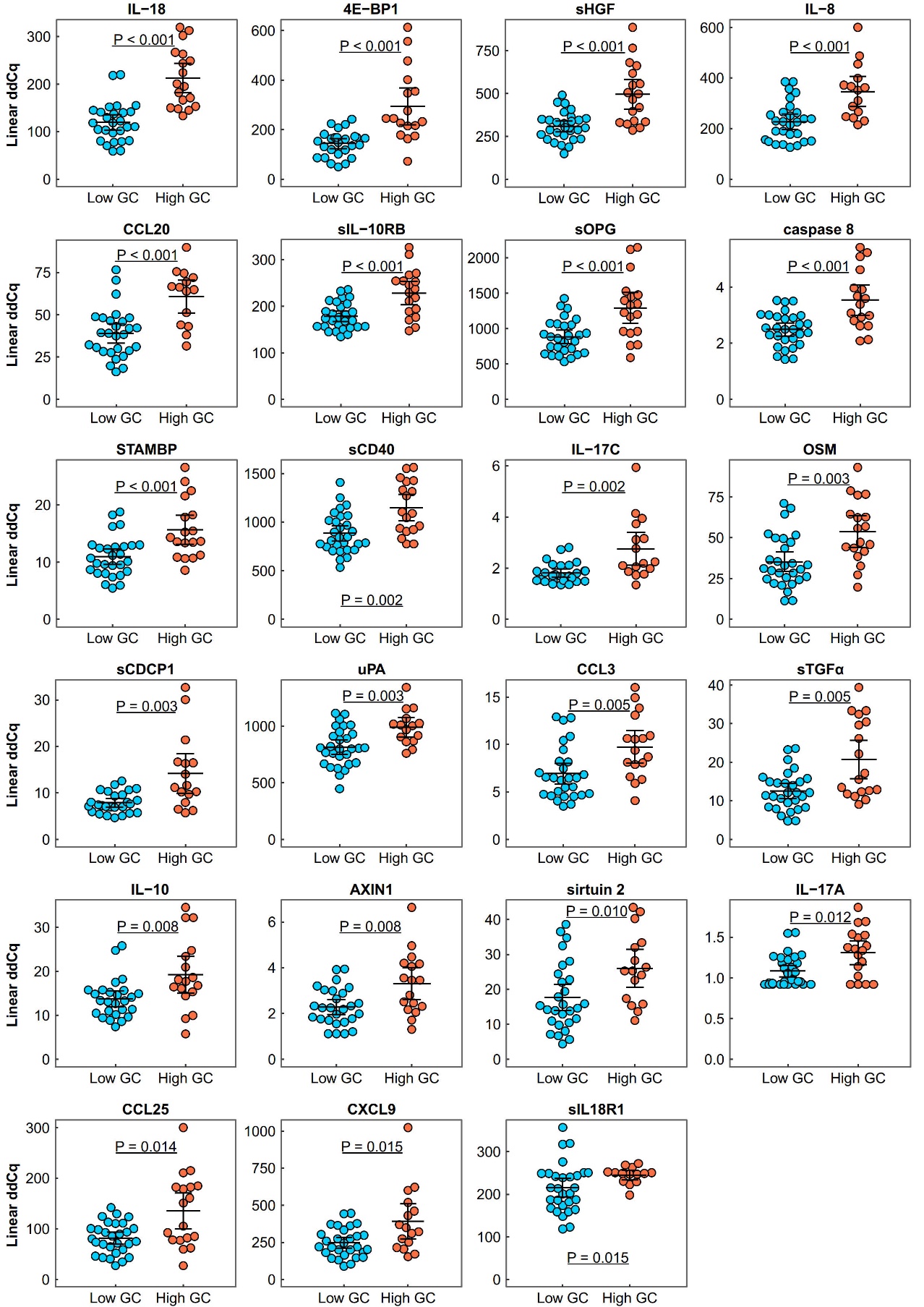


**Figure S2: Correlation of serum protein levels with cumulative GC dose within active RA.**

Blue: low GC group; red: high GC group.


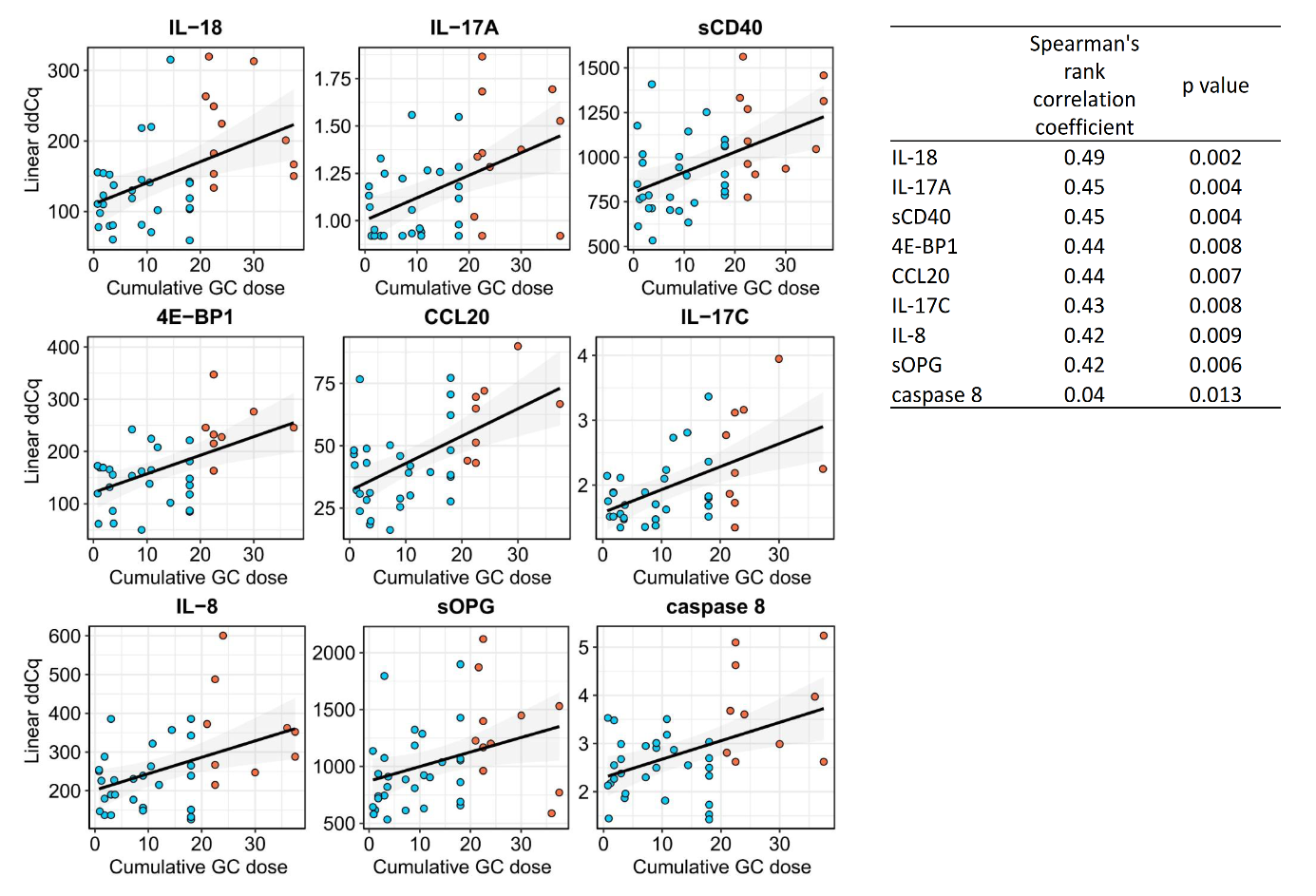


**Figure S3: Correlation matrix of clinical parameters of patients with RA.**

DAS28: 28-joint Disease Activity Score using C-reactive protein; GC: glucocorticoid; HAQ: health assessment questionnaire; SDAI: Simple Disease Activity Index.


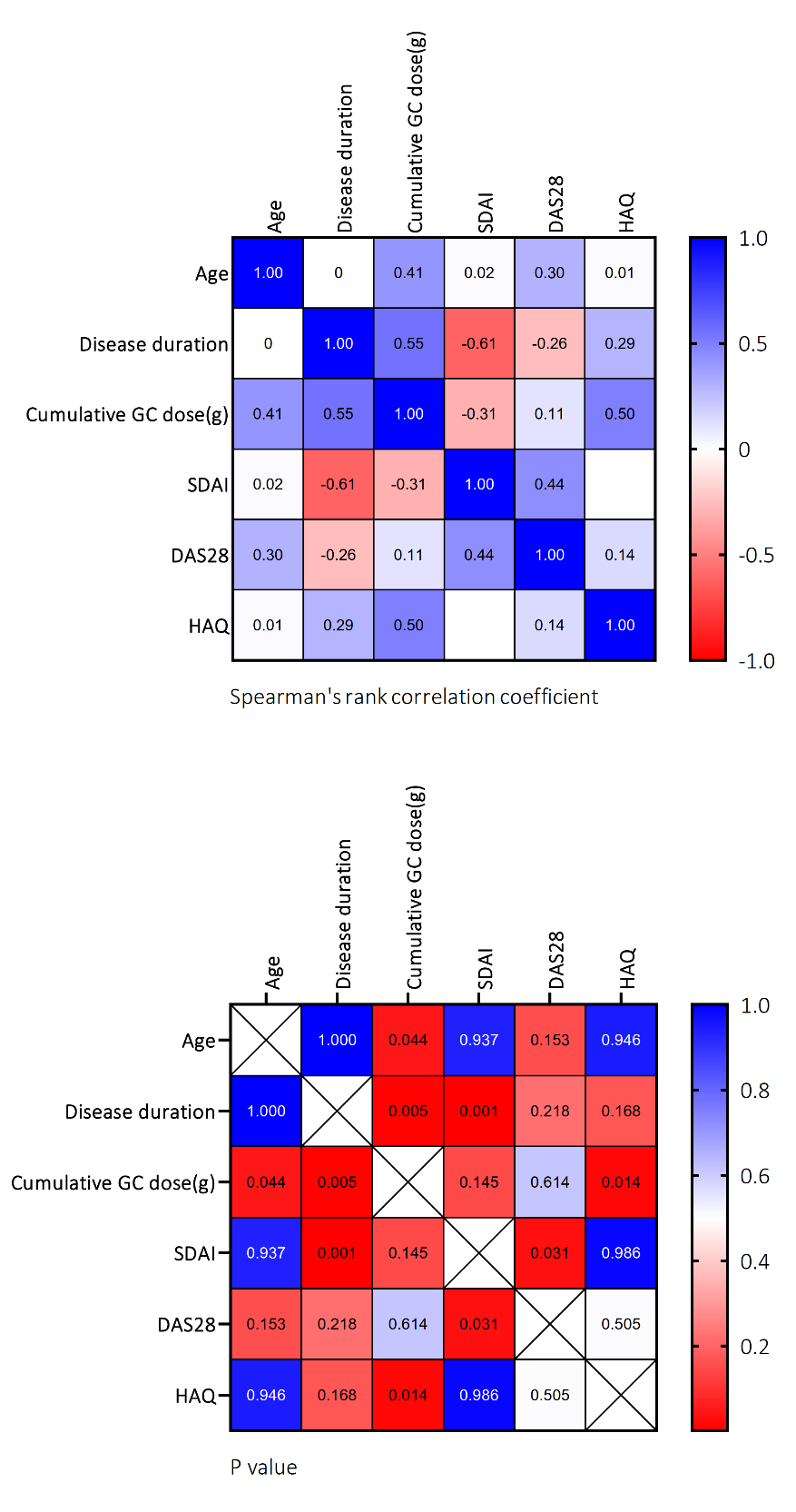


**Figure S4: Serum proteins associated with RA. Deregulated serum proteins associated with RA when compared to control group of healthy individuals, presented as log2 fold change (FC) of group medians.**

**
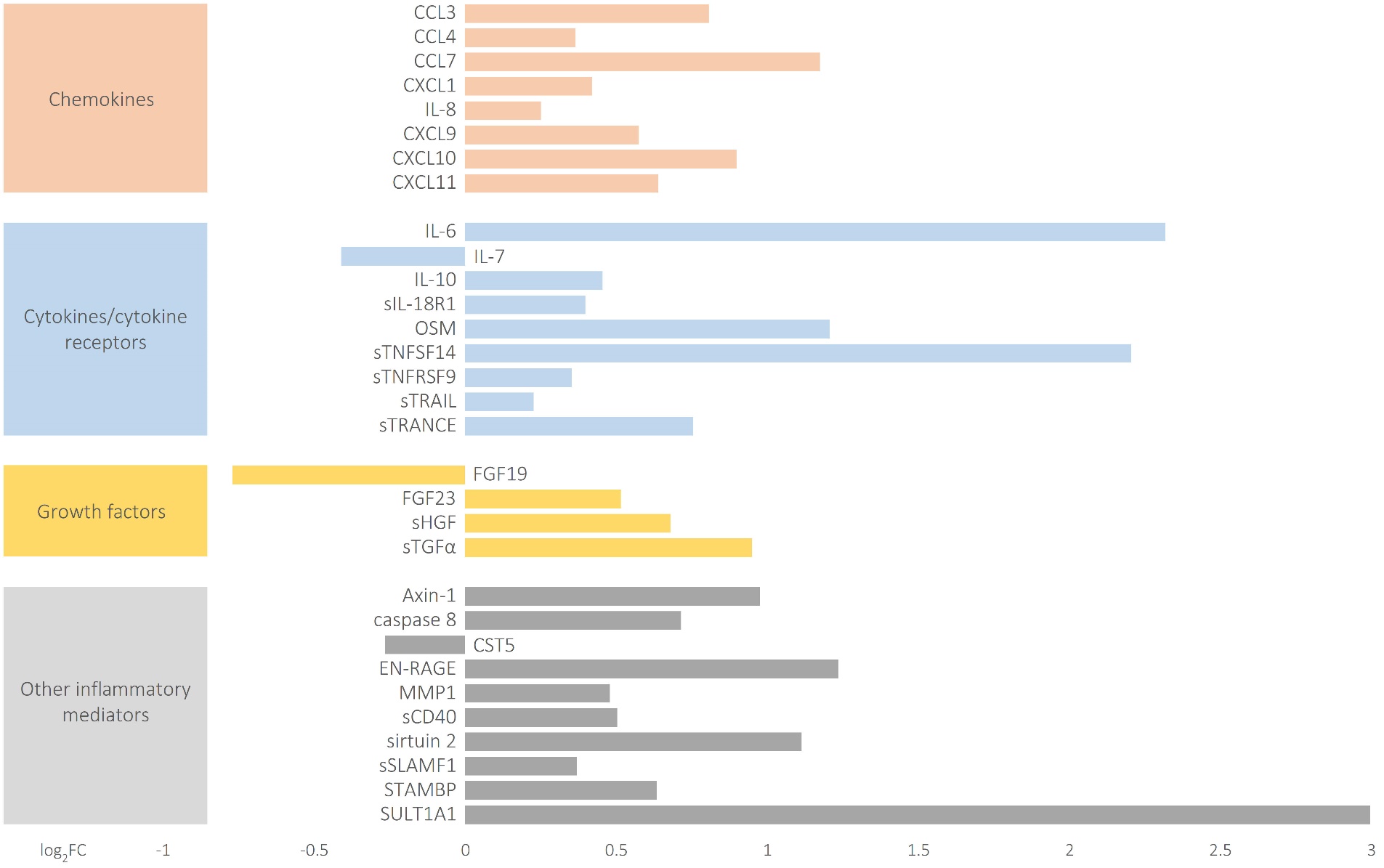
**
